# Advanced silk materials for musculoskeletal tissue regeneration

**DOI:** 10.3389/fbioe.2023.1199507

**Published:** 2023-05-02

**Authors:** Kexin Nie, Sicheng Zhou, Hu Li, Jingyi Tian, Weiliang Shen, Wenwen Huang

**Affiliations:** ^1^ Centre for Regeneration and Cell Therapy, The Zhejiang University—University of Edinburgh Institute, Zhejiang University School of Medicine, Zhejiang University, Hangzhou, China; ^2^ Department of Orthopedics of the Second Affiliated Hospital, Zhejiang University School of Medicine, Zhejiang University, Hangzhou, China; ^3^ Dr. Li Dak Sum and Yip Yio Chin Center for Stem Cells and Regenerative Medicine, Zhejiang University School of Medicine, Zhejiang University, Hangzhou, China

**Keywords:** silk, recombinant protein, processing, material modification, tissue regeneration, musculoskeletal system, bone, cartilage

## Abstract

Musculoskeletal diseases are the leading causes of chronic pain and physical disability, affecting millions of individuals worldwide. Over the past two decades, significant progress has been made in the field of bone and cartilage tissue engineering to combat the limitations of conventional treatments. Among various materials used in musculoskeletal tissue regeneration, silk biomaterials exhibit unique mechanical robustness, versatility, favorable biocompatibility, and tunable biodegradation rate. As silk is an easy-to-process biopolymer, silks have been reformed into various materials formats using advanced bio-fabrication technology for the design of cell niches. Silk proteins also offer active sites for chemical modifications to facilitate musculoskeletal system regeneration. With the emergence of genetic engineering techniques, silk proteins have been further optimized from the molecular level with other functional motifs to introduce new advantageous biological properties. In this review, we highlight the frontiers in engineering natural and recombinant silk biomaterials, as well as recent progress in the applications of these new silks in the field of bone and cartilage regeneration. The future potentials and challenges of silk biomaterials in musculoskeletal tissue engineering are also discussed. This review brings together perspectives from different fields and provides insight into improved musculoskeletal engineering.

## 1 Introduction

Musculoskeletal diseases (MSDs) are a group of complex conditions affecting bones, muscles, joints, and other connective tissues in the body. MSDs may result in acute and chronic pain, constrain mobility and dexterity, and cause burden in other health domains. The substantial impact of impaired musculoskeletal health, characterized by morbidity and mortality, is now globally recognized. Among various types of mammalian musculoskeletal tissues, bone and cartilage display a limited self-repair capacity, especially for large-scale defect repair. The intrinsic repair capacity also varies with individual differences, such as age, metabolic condition, and disease severity (Briggs et al., 2016). Currently, metallic cranial fixation devices and systems remain the gold standard for bone defect repair because of their outstanding mechanical properties (Broaddus et al., 2002; Farah et al., 2016; Khader and Towler, 2016). However, there exist some limitations in consideration of the numerous disadvantages of metal alloys including extreme stiffness, the potential risk of infection and immunological rejection, and second surgical removal (Imola et al., 2001; Zhang et al., 2012b). To overcome these limitations, resorbable devices that are composed of poly-L-lactic acid and polyglycolic acid have been utilized to reduce second surgical removal and promote bone remodeling. However, conventional resorbable materials are generally related to mild to severe inflammation under the effect of degradation products, osteolysis, and incomplete bone reconstruction (Eglin and Alini, 2008b). On the other hand, the innate self-repairing capacity of cartilage is limited due to its avascular and aneural nature. Currently, conventional clinic treatments for cartilage restoration include surgical procedures and conservative treatments, of which the common ones are debridement and continuous lavage under arthroscope, autologous chondrocyte implantation (ACI), and other treatments (Hutmacher, 2000; Emans et al., 2010; Abdel-Sayed and Pioletti, 2015). However, their boundedness still remains to be resolved, including donor deficiency, complications during the donor site recovery, insufficient durability of implants, and low transplantation success rate (Cheng et al., 2018). Despite recent advances in surgical and medication treatments, it remains challenging to fully restore the function of damaged musculoskeletal tissues. Therefore, designing advanced functional biomaterials which can provide support, promote healing, and restore function for musculoskeletal systems has been paid increasing attention (Vinatier et al., 2009; Wu et al., 2014; Docheva et al., 2015; Wei et al., 2021).

Among the biomaterials and devices for musculoskeletal tissue repair and regeneration, silk biomaterials are regarded with great potential owing to their unique mechanical robustness, chemical versatility, tunable biodegradation rate, and favorable biocompatibility (Vepari and Kaplan, 2007). Silks are a family of highly expressed fibrous proteins that are secreted and used externally by insects and spiders. Natural silkworm silk fibers are strong, flexible, and lightweight, making them ideal for conventional raw materials in textile industries. In recent decades, bioactive silk materials have gained particular interest as a family of emerging biomaterials in the field of tissue engineering (Huang et al., 2018). The main motivations for using silks in the design of bioactive medical materials are their versatility, along with their well-known biocompatibility, biodegradability, low immunogenicity, and availability in large-scale production. Silk proteins are mainly composed of non-reactive amino acids, such as glycine and alanine, and a relatively small percentage of reactive amino acids, such as serine, cysteine, and tyrosine (Huang et al., 2018). The presence of reactive amino acids in silks offers active sites for chemical modifications, which serve as a simple route for controlling protein structure, property, and function to facilitate musculoskeletal system regeneration (Murphy and Kaplan, 2009). With the emergence of genetic engineering techniques, modular silk templates have been further emulated and expanded with other functional motifs to introduce new advantageous biological properties (Zhou et al., 2015). Synergetic integration of multi-scale simulation at the early stages of material design also provides a more rapid solution to build bioactive materials from the molecular level (Huang et al., 2017). In addition to silk sequence alteration and modifications, silk biomaterials have also been reformed into a variety of biomaterials formats to recapitulate artificial cell niches using advanced bio-fabrication technology (Huang et al., 2018). The characteristic features in silk biomaterials, including tunable mechanical stiffness, surface nanopatterning, and surface chemistry can strongly influence cell behaviors, including cell adhesion, proliferation, migration, differentiation, and cell signaling (Sofia et al., 2001; Altman et al., 2003; Panilaitis et al., 2003; Kim et al., 2005; Wang et al., 2005). Due to these advantages, silk has become a suitable candidate for biomedical applications, especially in bone and cartilage regeneration.

**FIGURE 1:**
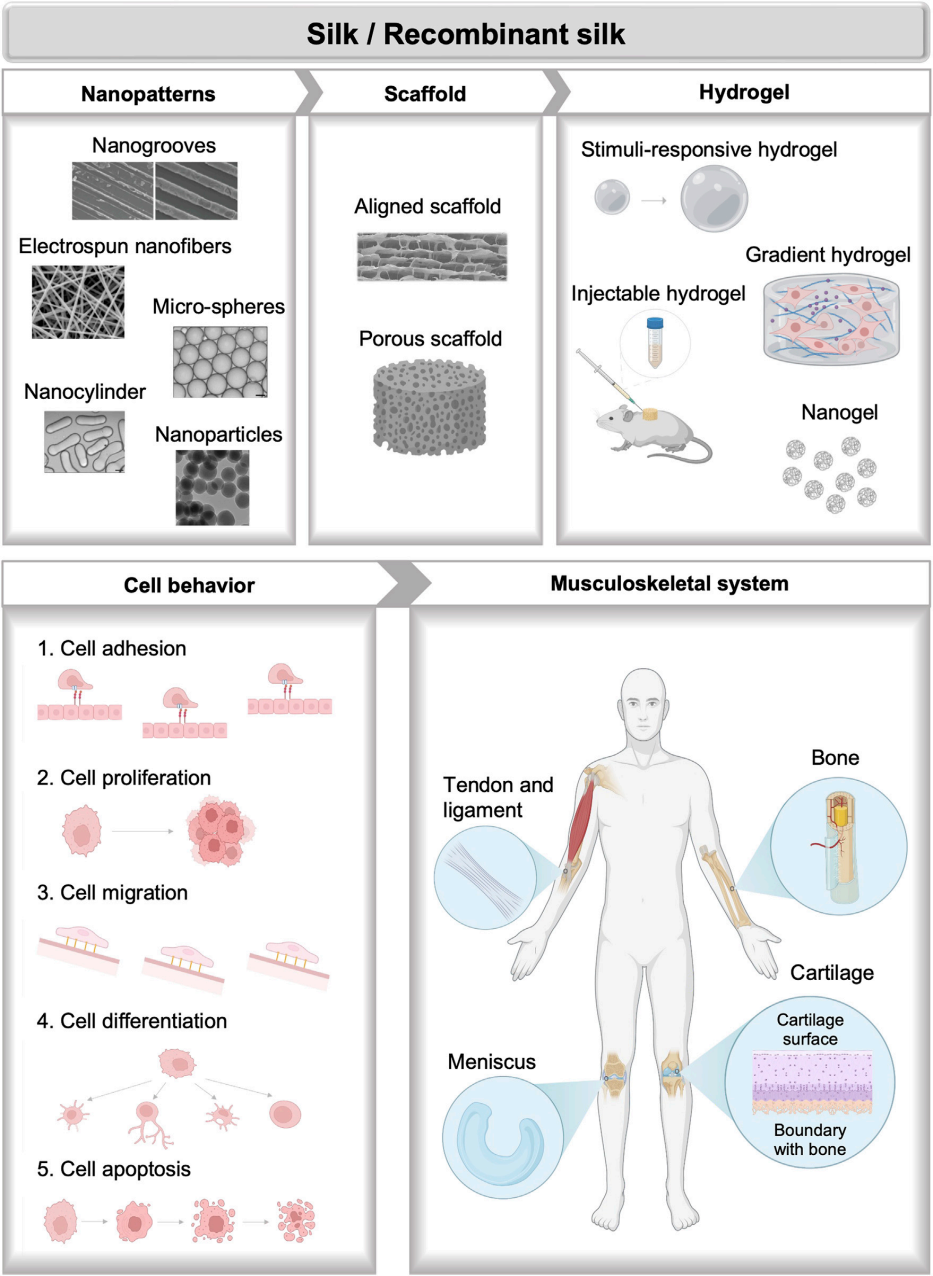
Bioactive natural and recombinant silk materials for musculoskeletal tissue regeneration.

Recent reviews have extensively discussed the advances and applications of silks in tissue engineering, which are usually focused on the discussion of different silk formats, including silk-based films, sponges, electro-spun and wet-spun fibers and yarns, hydrogels, particles, etc., along with the specific fabrication and functionalization methods. Applications of silk-based materials engineering in a broad spectrum of tissues including vascular, bone, neural, skin, cartilage, tendons, ocular, cardiac, and bladder tissues have been summarized based on the discussion of formats diversification of silk-based biomaterials (Kundu et al., 2013). Diverse chemical and physical methods for the fabrication of bio-mimetic architecture of silk-based biomaterials along with their biomedical applications are also highlighted (Kundu et al., 2014). Discussions of silks especially for musculoskeletal tissue engineering also gained a wide range of interest. Morphological diversification of silk-based material formats for musculoskeletal tissue engineering including films, electro-spun fibers, hydrogels, 3-D porous scaffolds, and particles are of particular interest (Ma et al., 2018). Besides, processing methods of silks such as freezing-drying, salt leaching, gas foaming, electrospinning, and compositing for cartilage regeneration are also discussed (Cheng et al., 2018). A review of different formats of spider silk-based materials applied in bone and cartilage regeneration is summarized, however, silks from other sources like silkworms are not included (Bakhshandeh et al., 2021). This review seeks to highlight recent basic and translational progress in silk biomaterials with applications in the field of musculoskeletal tissue repair and regeneration (Figure 1). In the beginning, a brief description of bone and cartilage biology, and the current synthesis method of silks are presented. Next, a detailed summary of recent progress in engineering natural and recombinant silk biomaterials is presented, with an emphasis on applications in musculoskeletal tissue engineering. At the end, the future potentials and challenges are discussed. This review differentiates itself from previous documents by focusing on the novel design of silk-based biomaterials including natural and recombinant silk biomaterials from diverse sources for cell niche mimicking along with their applications in musculoskeletal tissue engineering. It also highlighted the special properties including the soft and adaptable properties, mechanical performance, gradient biochemical and physical signal distribution, and functional motifs recordability of different silk formats through advanced bio-fabrication and genetic engineering methods.

## 2 Biology and regeneration of musculoskeletal systems

The ultimate goal in musculoskeletal tissue engineering is to fully restore the function of musculoskeletal tissues or organs. To achieve this goal, bioinspired tissue engineering materials are designed with similar mechanical stiffness, micro-nanostructure, chemical component, as well as cellular components of biological tissues to mimic the natural formations of musculoskeletal systems. These biomimetic approaches involve building complex structures of different tissue types with the aid of regenerative biomaterials. Therefore, understanding the biology of musculoskeletal systems, including structure, mechanics, and tissue formation, is fundamental in musculoskeletal tissue engineering.

### 2.1 Bone biology

**FIGURE 2:**
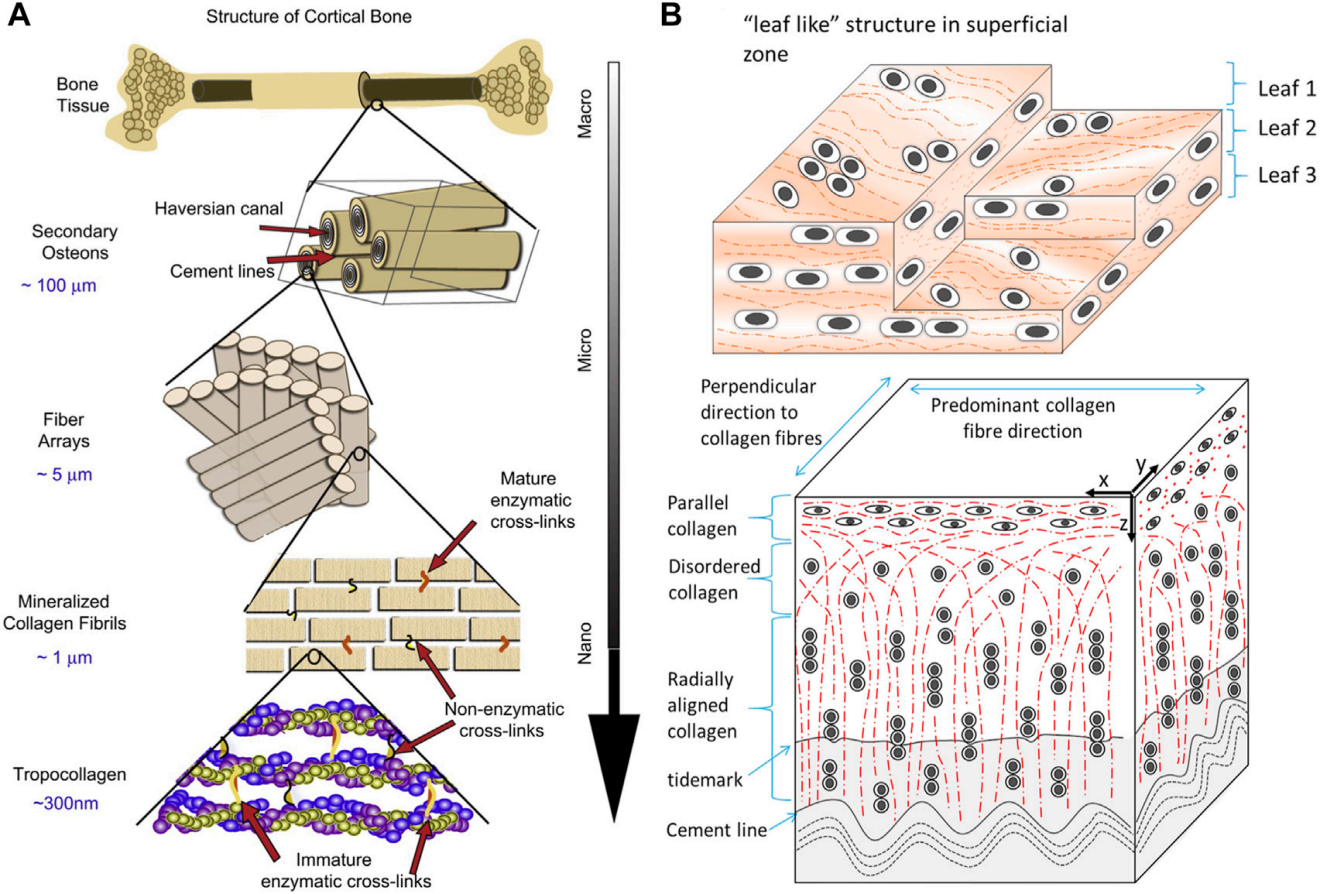
Schematic diagrams of the hierarchical structure of **(A)** human cortical bone and **(B)** cartilage. Adapted with permission from ref (Barth et al., 2011; Mansfield et al., 2015). Copyright 2011 Elsevier, 2015 Elsevier.

Bone, a metabolically active and mineralized connective tissue, supports locomotion, protects soft tissue, harbors bone marrow, stores calcium and phosphate, and regulates blood pH. Cortical and cancellous bone are the two main structural types of bone. They primarily differ in density, porosity, and metabolism which influence their function and physiology. As a compact bone, cortical bone is dense and solid, providing bending resistance and compressive strength (Figure 2A) (MacIntosh et al., 2008; Barth et al., 2011). In contrast, cancellous bone is spongy, porous, and precisely arranged, allowing deformation and absorption of loads. The internal structure and external morphology are in accordance with their functions and biological mechanics, and can be dynamically adjusted (Florencio-Silva et al., 2015). In healthy adults, bone contains about 70% inorganic minerals whose main content is hydroxyapatite (HA) [Ca_10_(PO_4_)_6_(OH)_2_] and about 30% organic matrix mostly composed of type I collagen (Brodsky and Persikov, 2005; Clarke, 2008). The inorganic mineral stores almost all calcium and phosphorous in the body (Florencio-Silva et al., 2015). While organic matrix gives elasticity and flexibility to tensile forces, inorganic mineral provides mechanical rigidity and resistance to compressive forces of bone. Hydroxyapatite crystals, measuring less than 20 nm, within collagen fibrils can activate the expression of alkaline phosphatase to promote the ordered deposition of minerals (Clarke, 2008; Bhattacharjee et al., 2017).

Bone tissue possesses the intrinsic capacity to completely regenerate in response to injury (Dimitriou et al., 2011). In terms of the majority of fractures, the bone heals without scar tissue formation and the fresh bone becomes indistinguishable from nearby healthy bone (Einhorn, 1998). However, bone regeneration may be impaired when facing oral and maxillofacial surgery, large quantity defects, avascular necrosis, and so on. Insufficient regeneration may lead to delayed union or fracture non-union (Dimitriou et al., 2011).

### 2.2 Cartilage biology

Cartilage is a resilient and elastic connective tissue found in joints, rib cages, noses, airways, intervertebral discs, and so on. Cartilage can absorb impact, lubricate the joint, and form or support some organs (Krishnan and Grodzinsky, 2018). Cartilage tissue contains a low density of specialized cells, chondrocytes, that synthesize and secrete ground substance and extracellular matrix (ECM) which are rich in proteoglycan and collagen (Figure 2B) (Mansfield et al., 2015; Naba et al., 2016). Cartilage can be classified into three types: hyaline cartilage, fibrocartilage, and elastic cartilage. Hyaline cartilage, containing abundant type II collagen fibrillar networks, is the most common form and is mainly found on the articular surface. It enables the movement of joints by providing lubrication and reducing friction. Fibrocartilage is primarily found in the intervertebral discs for its resilience and elastic cartilage makes up the external ears and larynx (Umlauf et al., 2010).

In contrast to bone tissues, cartilage lacks vascular, neural, and lymphatic systems, which results in the slow turnover of ECM and the incapability of vascular system-invoked reparative processes. Consequently, cartilage damage is hard to self-heal (Endisha et al., 2018). Cartilage defects are classified into two types: partial (chondral) or full thickness (osteochondral), according to the depth of the lesion. Once cartilage defects occur, the limited self-repair capability of cartilage tissue is insufficient for regeneration.

## 3 Silk: materials overview

### 3.1 Native silk

**FIGURE 3:**
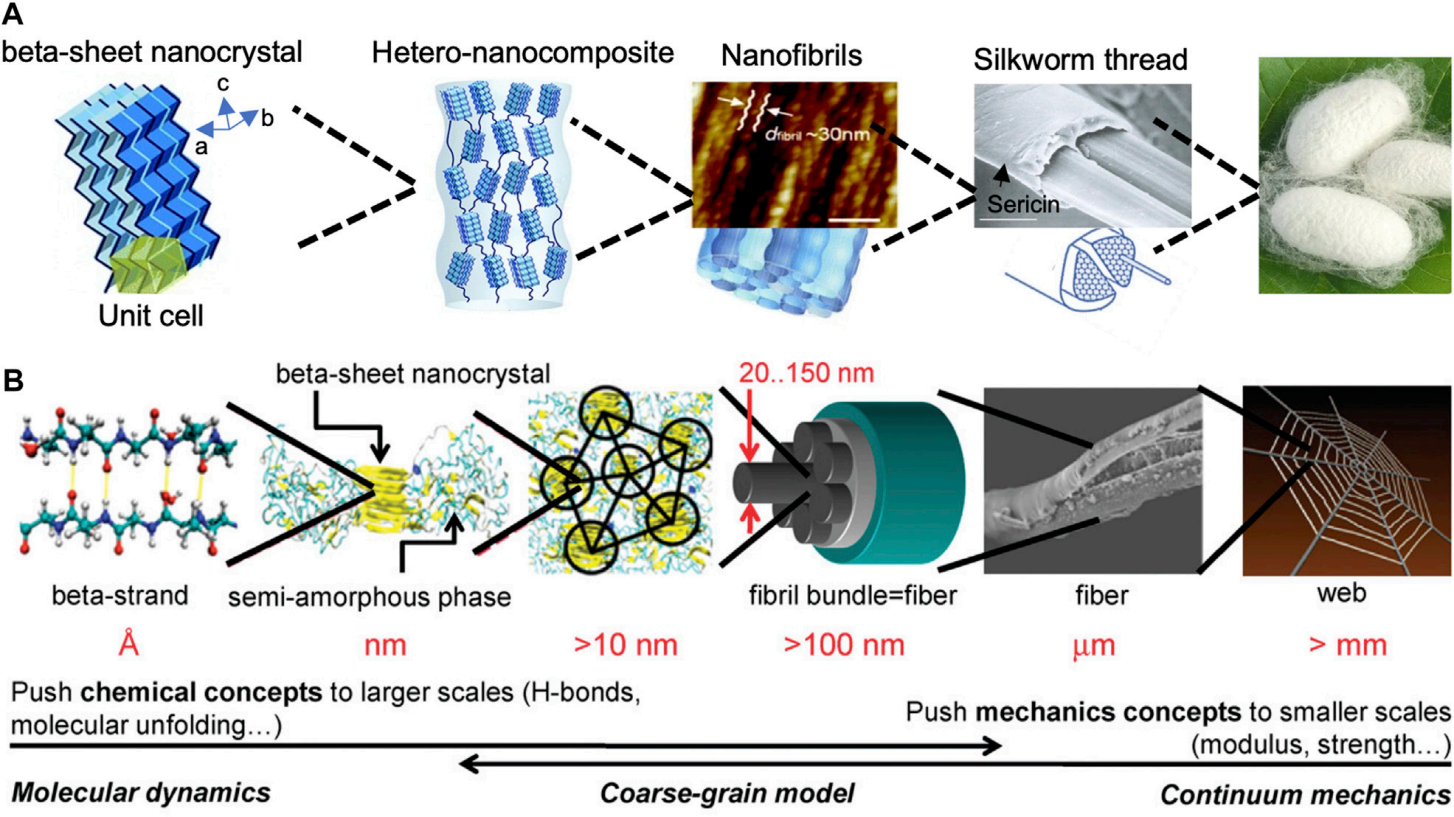
The hierarchical structure of **(A)** silkworm silk and **(B)** spider dragline silk fibers. Adapted with permission from ref (Giesa et al., 2011; Xu et al., 2014). Copyright 2014 Royal Society of Chemistry.

Silk protein is secreted from the gland of silk-producing arthropods, such as spiders, silkworms, and bees (Farokhi et al., 2018). Among various types of native silks, *Bombyx mori* silkworm silks have been widely investigated in tissue engineering, because of their remarkable mechanical properties, biocompatibility, biodegradability, non-cytotoxic, and suitable processability. *B. mori* silk consists of a core-shell structure with silk fibroin (SF) as the inner core and sericin as the outer coating (Figure 3A) (Rockwood et al., 2011; Huang et al., 2018; Pei et al., 2021). Sericin in silkworm silks is usually removed at the first step of silk material fabrication as it can elicit an allergreactionsion (Perez-Rigueiro et al., 2002). After the degumming process to remove sericin, SF can be directly processed into functional material forms, such as films, hydrogels, porous sponges, and nanoparticles for biomedical applications (Rockwood et al., 2011). SF consists of 3 major proteins: a light chain (∼26 kDa), a heavy chain (∼391 kDa), and a glycoprotein, P25 (Rockwood et al., 2011). The light chain is composed of a non-repetitive sequence with stronger hydrophilicity and less differentiated amino acids. The heavy chain is composed of repetitive hydrophobic blocks of (GA)nGX of varying lengths and arrangements, which tends to form β-sheet crystalline domains (Zhou et al., 2001). The hydrogen-bond β-sheet content in SF biomaterials influences the mechanical robustness remarkably (Gosline et al., 1999; Sponner et al., 2007; Hedhammar et al., 2008; Keten et al., 2010).

Owing to the mechanical robustness, tunable biocompatibility, and controllable enzymatic biodegradation, new biomedical materials based on silk have been explored. Perrone et al. designed new silk-based screws which exhibit high biocompatibility and promote bone remodeling (Perrone et al., 2014); a silk-based cranial fixation system has been established for skull bone fixation (Liu et al., 2018). Also, silk has been utilized in the form of 3D-printed SF hydrogel and SF scaffolds in cartilage defect repair (Chen et al., 2020; Hong et al., 2020). Furthermore, native SF materials have shown extraordinary merits as a carrier for bioactive growth factors and drug delivery such as Tanshinone IIA and microRNAs, which has been demonstrated in the research of Hong et al. and Chen et al. (Sui et al., 2018; Chen et al., 2020). Various studies have proved silk materials effective in cell survival, migration, proliferation, and differentiation, and these advantages make SF materials suitable for bone and cartilage regeneration. However, the osteogenic capacity is limited, especially in large bone defect repair (Farokhi et al., 2018). Hence, blending SF and other materials offer a strategy to combine the useful advantages of both materials for improved bone and cartilage regeneration. Shi et al. (2017) developed functionally and structurally optimized SF/gelatin scaffolds, which provided a benign microenvironment supporting BMSC proliferation, differentiation, and ECM production. Besides, scaffolds composed of SF and other materials such as nanohydroxyapatite, carbon nanofiber, and cellulose have been designed and evaluated in bone and cartilage repairing (Shen et al., 2016; Naskar et al., 2017; Begum et al., 2020). Furthermore, appropriate bioactive molecules such as stromal cell-derived factor-1 (SDF-1) and bone morphogenetic protein-2 (BMP-2) and cells including human mesenchymal stem cells (hMSCs) are loaded at SF composites to further improve differentiation potential (Shen et al., 2016; Begum et al., 2020). Overall, SF and composite SF materials have served as ideal biomimetic materials for bone and cartilage regeneration.

### 3.2 Recombinant silk

The rational design of recombinant proteins has emerged as a powerful strategy for investigating the intricate relationships between protein sequence, structure, and function, offering a “bottom-up” approach for protein-based biomaterial design. Complex protein systems can be deconstructed into fundamental functional modules that govern structural motifs, providing insights into biomaterial function. Recent advances in genetic engineering have paved the way for the production of monodisperse and well-defined silk-based proteins, which serve as a fundamental building block for fabricating biomaterial systems that closely mimic the biological and mechanical properties of native tissues. Silk can be precisely tailored to meet the specific requirements of musculoskeletal tissue engineering materials through a diverse range of techniques, including the incorporation of non-natural amino acids, the introduction of specific structural domains and functionalized sequences, the development of hybridization strategies, and the generation of stimuli-responsive systems. The synergistic combination of structuring and functionalization of silk proteins presents innovative approaches, such as the design of novel drug delivery systems, biomineralized surfaces, tissue regeneration matrices, and mechanical support structures, which hold great promise in addressing the current challenges in musculoskeletal tissue engineering.

Spider silks are remarkable biomaterials when considering their light density and excellent mechanical properties due to their hierarchical structures (Figure 3B) (Huang et al., 2011). However, harvesting spider silk from natural sources is challenging, because spiders are solitary and territorial animals. One way to produce spider silk proteins in large quantities is through the expression of natural spider silk genes in different host organisms, such as prokaryotes, eukaryotes, and even transgenic animals (An et al., 2015; Huang et al., 2017). The construction of recombinant silk proteins includes three steps: plasmid construction, protein expression, and protein purification. To construct expression plasmids, a variety of cloning strategies have been used toward this goal, including step-by-step directional ligation, recursive directional ligation, and concatemerization (Dinjaski et al., 2018). Once the expression plasmid is successfully constructed, it is transformed into *E. coli* host for expression, then the expressed recombinant silk proteins are purified using affinity chromatography and size exclusion chromatography based on their affinity tags and molecular weight.

Among various types of spider silks, the dragline silk of the *N. clavipes* spider has served as a template model for basic structure-sequence-function relationship studies (Huang et al., 2011; Lin et al., 2015) and tissue engineering applications (Aigner et al., 2018). With the recent advance in genetic engineering techniques, silk proteins have been further optimized with functional peptide motifs to introduce new advantageous properties. *N.clavipes* dragline silks were cloned and expressed to include RGD cell-binding domains to introduce selective cell interactions (Bini et al., 2006). The recombinant silk-RGD proteins enhanced the differentiation hMSCs and bone-related outcomes, such as calcium deposition. Moreover, silk-VTK recombinant proteins have been designed to facilitate biomineralization, and support hMSCs growth and proliferation (Dinjaski et al., 2017). The recombinant silk proteins can be structurally and functionally engineered to meet the demands of different mechanical robustness, biodegradability, biocompatibility and the potential of osteochondral differentiation.

## 4 Engineering silks for musculoskeletal regeneration

Recent advances in the design and fabrication of nanostructured biomaterials have opened up new opportunities in musculoskeletal tissue engineering. Conventional strategies to manufacture silk-based materials include freeze-drying, gas foaming, and salt leaching (Rockwood et al., 2011). Recently, developments in micro- and nano-scale biotechnologies pave the way to integrate physical and biological cues in silk materials and devices for improved bone and cartilage regeneration (Funda et al., 2020). Compared with conventional strategies, advanced bio-fabrication and genetic engineering methods have displayed unique advantages, such as tunable porosity for growth factor delivery (Wang et al., 2012), mechanical enhancement in nano-scale, customized micro-nano patterning, gradient mechanical strength (Guo et al., 2017; Shi et al., 2017; Guo et al., 2020), and integration of biological cell niche in materials design (An et al., 2015; Zhou et al., 2015).

### 4.1 Soft and adaptable materials

Hydrogel is an efficient three-dimensional material for tissue engineering because of its high water content and flexibility, which allows it to mimic native ECM. Hydrogels are in general fabricated by crosslinking methods to combine two or more functional groups through covalent or non-covalent bonding to form a polymeric network, usually employed to complement each other in many features (Thakur et al., 2018; Krishnakumar et al., 2019). To endow ideal features to silk, it is essential to choose proper crosslinkers and approaches. Broadly, crosslinking approaches are classified into three main categories: physical, chemical and enzymatic crosslinking methodologies (Krishnakumar et al., 2019). Physical crosslinking is a safe, inexpensive, non-toxic and biocompatible procedure with the formation of β-sheets, including UV irradiation, shear force, and dehydrothermal treatment (Huang et al., 2018). For example, ethanol treatment of silk fibroin promoted the formation of β-sheets and enhance the mechanical strength (Shi et al., 2017). However, physical crosslinking lacks crosslinking degree and chemical bonds strength, and is relatively more difficult to control the reaction kinetics (Thakur et al., 2018). In contrast, chemical crosslinking provides stronger bonds by connecting different substances with ionic and covalent bonds. In addition, chemical crosslinking makes it possible to introduce some functional groups which are absent in silk fibroin (Kim et al., 2018). For instance, glycidyl methacrylate (GMA) was covalently immobilized with silk fibroin to form pre-hydrogel. GMA donated a vinyl double bond that acted as the site of UV-crosslinking (Kim et al., 2018). On the other hand, the products should be washed to get rid of the toxic residual crosslinkers, but the residues would be left more or less (Thakur et al., 2018). Enzymatic crosslinking features with high crosslinking efficiency and mild reaction conditions with the help of catalysts. Significantly, a large quantity of the catalysts has no cytotoxicity or immunogenicity (Krishnakumar et al., 2019), and the reaction can be easily controlled due to the sensitivity of enzymes to external conditions such as temperature and pH. Hasturk et al. (2020) developed the HRP/H2O2 mediated crosslinking methods to form SF composite hydrogels and successfully meliorated gelation kinetics and bioactivity.

**FIGURE 4:**
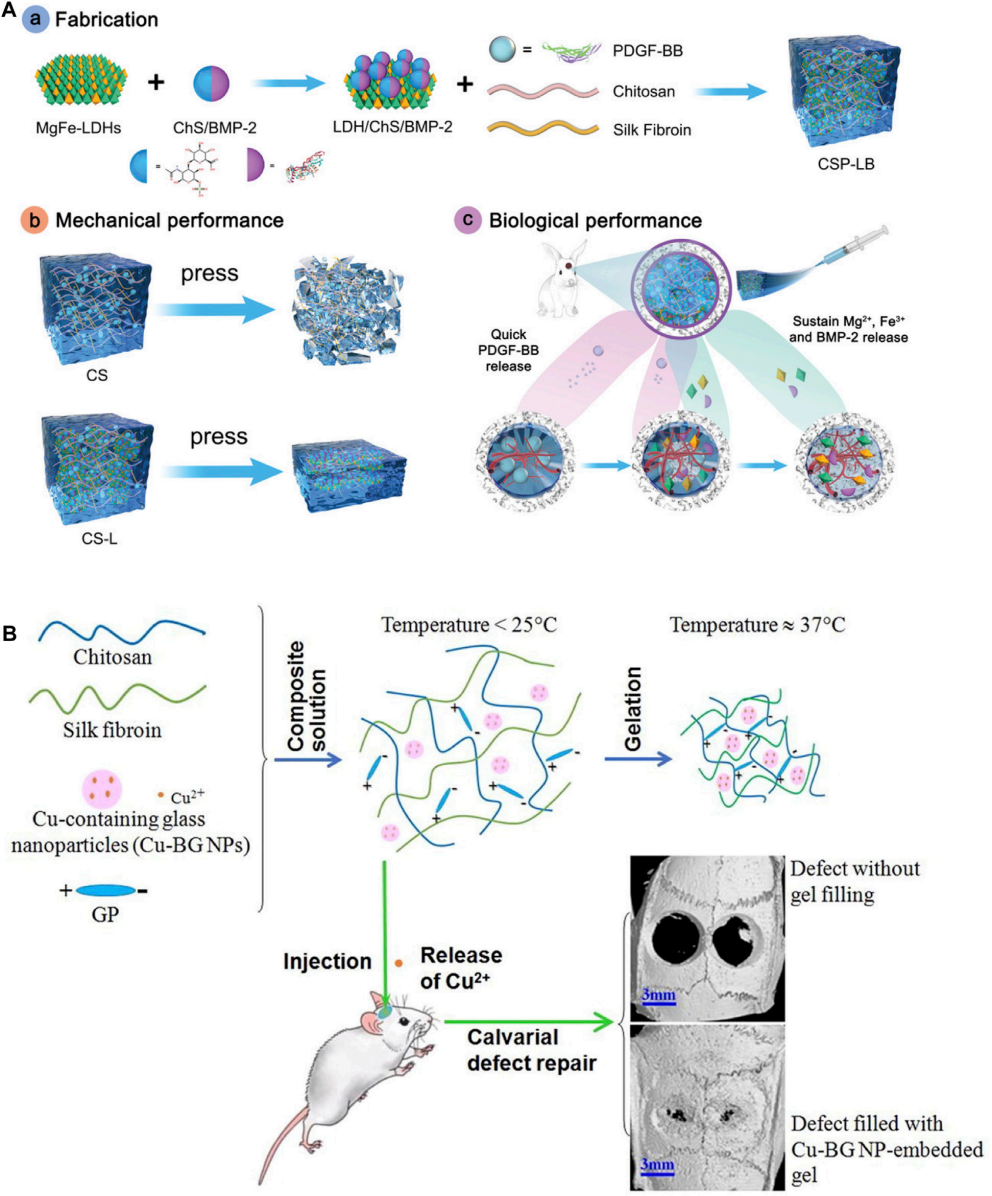
Injectable silk protein-based hydrogels. **(A)** Angiogenic PDGF-BB loaded chitosan/silk fibroin (CS) hydrogel incorporated with osteogenic BMP-2-functionalized MgFe-LDH. **(B)** Injectable chitosan (CH)/silk fibroin (SF)/glycerophosphate (GP) composite hydrogels triggered by temperature. Adapted with permission from ref (Wu et al., 2019; Lv et al., 2022). Copyright 2019 Elsevier, 2022 WILEY-VCH.

Compared to traditional hydrogels, injectable hydrogels can fill irregular defects and form stable 3D polymer networks *in situ*. This non-invasive injection method can avoid implantation surgery, which is convenient and efficient. Silk fibroin has been widely used for injectable hydrogel owing to its biocompatibility, controlled biodegradability and ease of manufacturing. Moreover, growth factors were usually incorporated into silk fibroin based injectable hydrogels. Growth factors were widely used in tissue engineering since they can inhibit or promote cellular adhesion, spreading, proliferation, differentiation, and gene expression. Growth factors can be added directly into the scaffolds during or after fabrication. They can also be delivered into scaffolds through some carriers like nanoparticles. Hydrogels were beneficial for carrying growth factors since their 3D structures can hold growth factors and maintain their stability. Moreover, hydrogels can control the release of drugs through diffusion or degradation mechanism. The co-delivery of angiogenic and osteogenic growth factors (GFs) has emerged as a promising strategy for bone regeneration. Silk fibroin, owing to its excellent biocompatibility and high-water content, has gained attention as an attractive carrier for GF delivery. Angiogenic platelet-derived growth factor-BB (PDGF-BB) loaded chitosan/silk fibroin (CS) hydrogel was incorporated with osteogenic bone morphogenetic protein-2 (BMP-2)-functionalized MgFe-2D layered double hydroxide (MgFe-LDH) for repairing bone defects (Figure 4A) (Lv et al., 2022). The GF-loaded hydrogel demonstrated good injectability and rapid thermo-responsiveness below physiological temperature (37°C) due to the presence of silk fibroin and LDH. Furthermore, the sequential release of dual GFs was achieved, with the burst release of PDGF-BB from CS hydrogel and the sustained release of BMP-2 from MgFe-LDH. *In vivo* tests revealed that this composite hydrogel can improve bone regeneration by considerably increasing bone volume and mineral density when compared to other groups. This temperature-responsive injectable hydrogel exhibited excellent gelation properties and sequential GF-releasing behavior, offering a promising minimally invasive approach for bone repair. In other research, silk nanofibers (SNF) hydrogel was obtained by pre-culture silk fibroin solution at 60°C, which was injectable using syringes by hand while remaining solid hydrogel state after injection. Deferoxamine (DFO) and bone morphogenetic protein-2 (BMP-2) were loaded on SNF and HA for introducing angiogenic and osteogenic cues. This injectable hydrogel mimics multiple biophysical and chemical cues including the fiber-matrix morphology, organic-inorganic chemical content, and regulating of growth factor, showing good bone regeneration (Cheng et al., 2020). Chitosan (CH)/silk fibroin (SF)/glycerophosphate (GP) composites were also fabricated as injectable hydrogel with thermal-triggered gelation at physiological temperature and pH (Figure 4B) (Wu et al., 2019).

### 4.2 Mechanical enhancement

Over the years, thermal processing technology has been used as a rapid and low-cost fabrication method in the plastic industry. However, under elevated temperatures, native silk fibroins with relatively high β-sheet content tend to degrade before melting, limiting the application of thermal remolding. Recently, Guo et al. (2020) have developed a novel, significantly more efficient fabrication method for thermoplastic molding silks into high-density and tough medical devices such as bone screws, plates, and ear tubes. Guo et al. reconstructed regenerated silk fibroins into amorphous silk nanomaterials (ASN) in powder form with low β-sheet content (<1.0%) and high random coil or helix content. These ASN underwent structural transitions into viscoelastic liquid at defined glass-transition temperature (Tg’), allowing the remolding procedure. Moreover, at higher processing temperatures (about 145°C) and pressure (632 MPa), β-sheet content would increase and ASN would self-assemble into more homogenous and densified bulk silk-based biomaterials such as bone screws. The whole procedure was time- and cost-effective and can be tuned to incorporate bioactive molecules at mild temperatures by adjusting the absorbed moisture content in silks. The bone screws implanted in rat femurs had no surficial inflammation, indicating their favorable biocompatibility (Guo et al., 2020).

**FIGURE 5:**
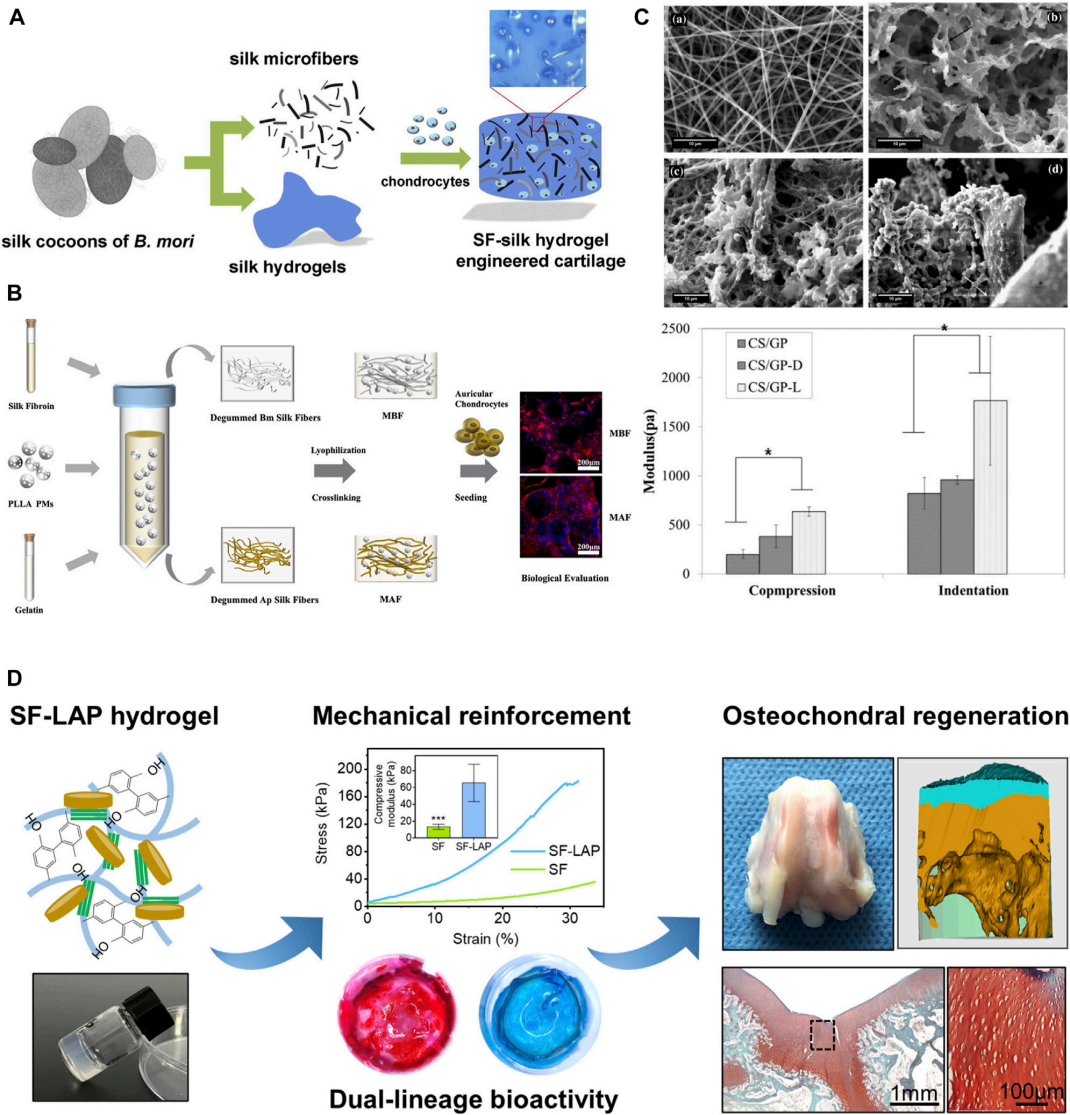
Mechanical reinforcement of silk protein hydrogels. **(A)** Silk microfiber reinforced silk hydrogels and their equilibrium and dynamic moduli with different silk fiber lengths. **(B)** Two kinds of silk fibers either from *Bombyx mori* (Bm) or *Antheraea pernyi* (Ap) and PLLA microspheres co-reinforced hydrogels for auricular cartilage tissue engineering. **(C)** SEM images of silk nanofiber (top left), chitosan/glycerophosphate hydrogels (CS/GP) (top right), degummed chopped silk fibers reinforced CS/GP (CS/GP-D) (bottom left), electrospun silk mats reinforced CS/GP (CS/GP-L) (bottom right) and their modulus. **(D)** Laponite (LAP) reinforced silk hydrogels for osteochondral regeneration. Adapted with permission from ref (Mirahmadi et al., 2013; Yodmuang et al., 2015; Yang et al., 2021; Zhang et al., 2021). Copyright 2013 Elsevier, 2015 Elsevier, 2021 Elsevier.

Hydrogels are regarded as having great potential owing to their high water content, three-dimensional cell culture capacity, tunable porous framework and robustness, and good biocompatibility. Mechanical reinforcements in hydrogels have also been achieved by using silk microfibers with other biomaterials to form a “steel bars reinforced concrete” structure (Figure 5). Since silk has high elastic modular and toughness, under loading, silk microfibers can be stretched and dissipate stress fast in the composite hydrogels and then achieve mechanical enhancement of silk protein composites. Silk microfiber-reinforced silk hydrogels were fabricated by mimicking the fiber and proteoglycan composite structure of biological cartilage. This hydrogel showed reinforced equilibrium moduli within the range of stiffness of native cartilage (Yodmuang et al., 2015). Two kinds of silk fibers either from *B. mori* or *A. pernyi* and PLLA microspheres co-reinforced hydrogels are also developed for auricular cartilage tissue engineering (Yang et al., 2021). Moreover, silk electrospun membranes can also reinforce the stiffness of the composites through layer-by-layer sandwich structures. It was reported that the electrospun silk mats reinforced chitosan/glycerophosphate hydrogels showed better robustness than degummed silk fibers (Mirahmadi et al., 2013). Furthermore, other fillers like nanoparticles can also reinforce the silk protein hydrogels (Zhang et al., 2021).

### 4.3 Gradient within materials

**FIGURE 6:**
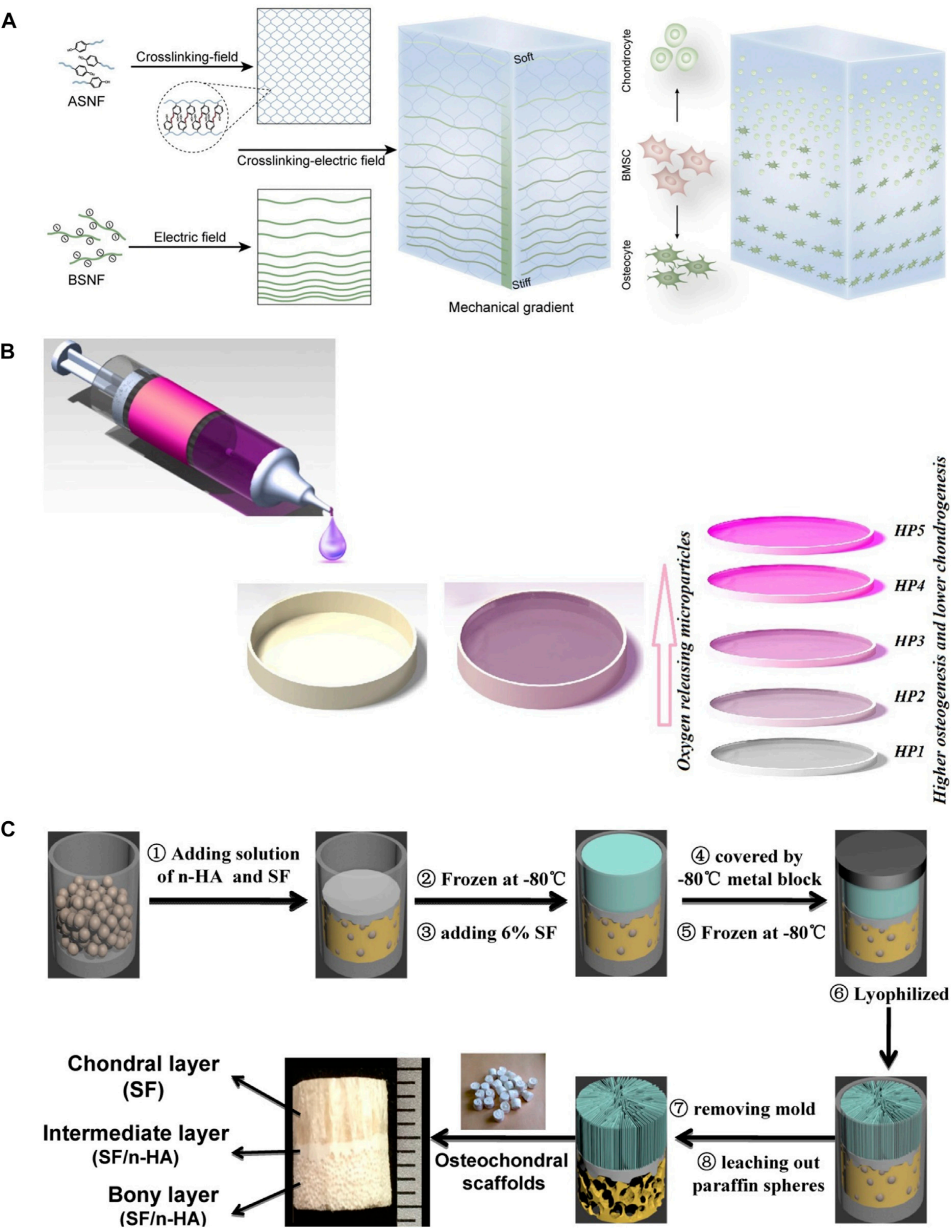
Mechanical gradient of silk protein hydrogels. **(A)** Electric field and gelation of ASNF-induced gradient distribution of BSNF. **(B)** Gradient distribution of polylactic acid (PLA) and calcium peroxide (CPO) microparticles in silk fibroin hydrogel through a gradient mixing chamber. **(C)** Integrated tri-layer osteochondral tissue engineering scaffold composed of the upper SF layer with oriented structure, dense intermediate SF/HA layer, and bottom SF/HA layer with porous structure through paraffin leaching. Adapted with permission from ref (Ding et al., 2014; Xu et al., 2020; Khorshidi et al., 2021) Copyright 2021 Elsevier.

Bone and cartilage have different biochemical components and mechanical stiffness. Therefore, integrated repair of osteochondral defects requires scaffolds with mechanical gradients simulating the native integrated structure of bone and cartilage. Recently, an electric field was used to form silk fiber reinforced mechanical gradient hydrogels. Beta-sheet rich silk nanofibers (BSNF) moved from cathode to anode in the matrix of amorphous silk nanofiber solutions (ASNF) under the driving force of electric field. When the ASNF gelation occurred, the formed aligned and gradient distribution of BSNF was solidified. By adjusting electric field intensity and gelation time of ASNF, the velocity of BSNF movements can be tuned and tunable mechanical gradient silk hydrogels can be obtained (Figure 6A) (Xu et al., 2020). Fabricating materials with gradient concentrations is another efficient way to induce gradient in biomaterials. Hydrogel/particle scaffold with a gradient of the oxygen-releasing microparticles were fabricated by adding polylactic acid (PLA) and calcium peroxide (CPO) microparticles in silk fibroin precursor through a gradient mixing chamber (Figure 6B) (Khorshidi et al., 2021). The increase in microparticle content promoted osteogenesis, whereas the parts with low microparticle content encouraged chondrogenesis of mesenchymal stem cells.

3D printing is an emerging bottom-up technology, by which 3D biomimetic architecture can be established. According to the predesign assisted with computer-aided design (CAD), custom microstructure can be obtained by printing materials layer by layer with a microstructure that is precisely controlled to the nanoscale (Zhu et al., 2016a; Włodarczyk-Biegun and Del Campo, 2017; Huang et al., 2018). Therefore, 3D printing can also be used for designing scaffolds with gradient architecture and mechanical signals. Digital light printing (DLP) is one of the widely used ways for creating layered scaffolds with synthetic complexes with desirable structures and ideal cell types in tissue or organ regeneration (Grogan et al., 2013; Lin et al., 2013; Zhu et al., 2016b; Zheng et al., 2016). Parathyroid hormone (PTH) grafted silk fibroin (SF-PTH) and methacrylic anhydride chemically modified silk fibroin (SF-MA) were mixed with gelatin methacryloyl (GM) as two bio-inks (Deng et al., 2021). Biphasic scaffolds composed of a layer of SF-PTH/GM hydrogel and a layer of SF-MA/GM hydrogel were fabricated using these two bio-inks through 3D bioprinting technology. SF-PTH/GM layer and SF-MA/GM layer exhibited different mechanical stiffness resulting in the mechanical gradient of these biphasic scaffolds. To further mimic the gradient mechanical and structural properties of osteochondral, a novel integrated tri-layered scaffold was created by combining directional crystallization and pore-forming technologies (Figure 6C) (Ding et al., 2014). The upper SF layer with a longitudinally oriented structure was generated by directional freezing, while the bottom layer with a subchondral bone mimics porous structure was composed of SF and HA and produced through paraffin-sphere leaching, and the intermediate layer was dense SF and HA that serves as the boundary between the two layers and enhances the mechanical strength of this gradient scaffold.

### 4.4 Encoding function motifs

The natural cellular niche can affect cellular activities through the stimulation of specific cell-materials interaction. Generally speaking, there are two classes of stimuli: physical cues, such as cell-adhesive sequences, and soluble cues, such as growth factors. Grafting and direct mixing of bioactive molecules into biomaterial matrices are conventionally used methods to incorporate biological cues. Mineralization is a process to introduce minerals into the bone structure, organized by osteoblasts after the deposition of the organic matrix, including calcium and phosphate. They will transform into crystalline calcium phosphate that provides stiffness. Bone remolding involves the formation of new bone and the absorption of old bone due to the orchestrated coordination among osteoblasts, osteoclasts, and osteocytes. It is essential for regulating calcium homeostasis and repairing the damaged bone. Silicification peptide grafted SF hydrogel was fabricated to mimic the natural gradient distribution of minerals in the extracellular matrix, and the mineralized SF hydrogels can induce osteogenic differentiation (Guo et al., 2017). The ion diffusion method was also utilized in biomimetic mineralization in silk hydrogels. Ca^2+^ was added to silk hydrogels for providing nucleation sites of hydroxyapatite crystals and further regulating their oriented growth (Jin et al., 2015). By varying the Ca^2+^ concentration and mineralization time, morphology and aggregation status can be controlled.

**FIGURE 7:**
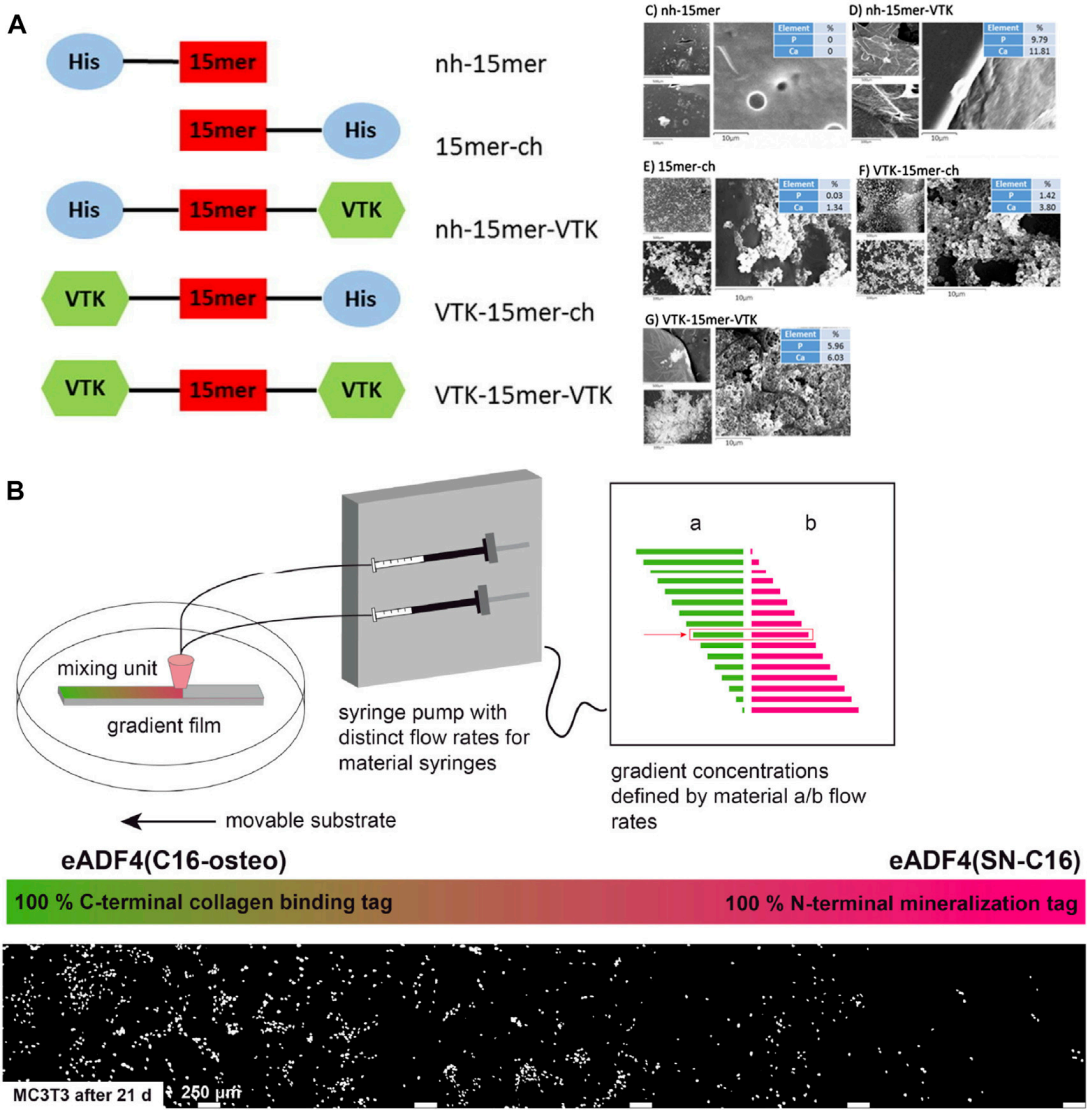
Mineralization and cell adherence of genetically engineered silks. **(A)** Silk-VTK fusion proteins and films for enhanced mineralization. **(B)** Spider silk–SIBLING hybrids showed enhanced calcium phosphate formation and preferentially adherence of pre-osteoblasts along the gradient toward the variant with a collagen-binding motif. Adapted with permission from ref (Dinjaski et al., 2017) (Neubauer and Scheibel, 2020) Copyright 2017 Elsevier, 2020 American Chemical Society.

Genetic engineering technology provides an advanced tool to integrate cell niches at the beginning of material design from the molecular level. VTKHLNQISQSY (VTK) has proven biomineralization properties with both bonelike minerals and hydroxyapatite (Addison et al., 2010). Dinjaski et al. combined silk domain (SGRGGLGGQGAGAAAAAGGAGQGGYGGLGSQGT)_15_, which is obtained from the common silk domain of *N. clavipes* dragline, and the hydroxyapatite binding domain VTKHLNQISQSY (VTK) to constructed silk-VTK fusion proteins. Five forms of silk-VTK fusion proteins were generated based on different positions and numbers of the VTK domain and were further made into films for mineralization test. Results showed the presence of the VTK domain enhanced osteoinductive properties up to 3-fold compared to the control (Figure 7A) (Dinjaski et al., 2017). Neubauer et al. engineered recombinant spider silk proteins with different mineralization and collagen binding tags. The variants were found to distinctly induce mineralization and increase the adherence of pre-osteoblasts (Figure 7B) (Neubauer and Scheibel, 2020).

## 5 Applications of silks in musculoskeletal regeneration

### 5.1 Bone tissue engineering

Bone defects normally arise from trauma, congenital malformations, tumor resection, and reconstructive surgery (Majidinia et al., 2018). In comparison to scar tissue that was incompletely repaired by collagen, entire healing of bone defect can be available due to cellular regeneration and matrix mineralization (Marx, 2007). Mesenchymal stem cells (MSCs) play a key role in the healing process. MSCs, with the characteristics of self-renewal and pluripotency, mainly exist in the bone marrow and can migrate around the clot, differentiate down osteogenic lineages, and activate downstream networks to facilitate bone induction and conduction (Shao et al., 2015; Majidinia et al., 2018).

**FIGURE 8:**
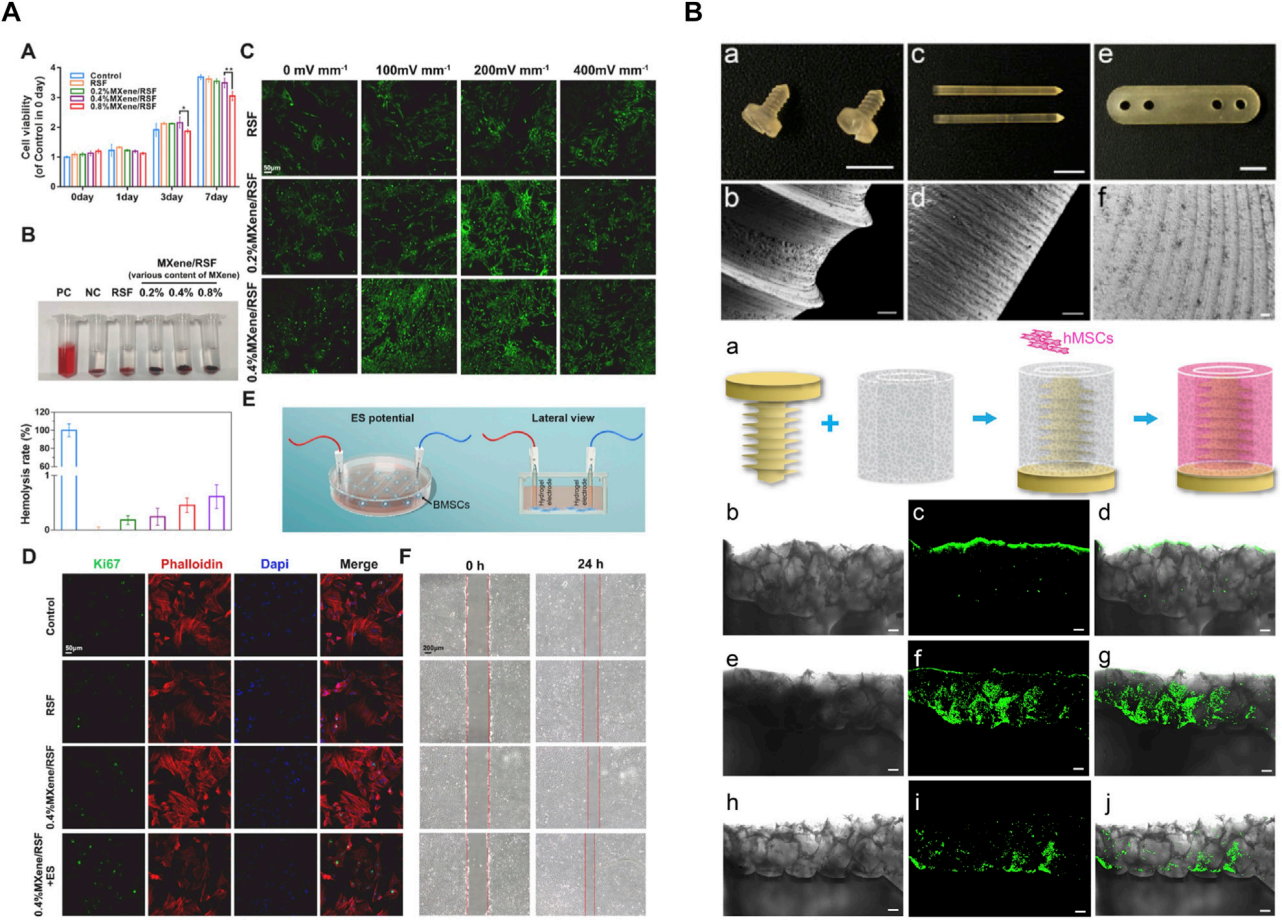
Silk hydrogels and devices for bone tissue regeneration. **(A)** SF-MXene hydrogel re-established the electrical microenvironment and promoted direct osteogenesis. **(B)** Silk-based orthopedic devices with osteoinductive functionality. Adapted with permission from ref (Li et al., 2016; Hu et al., 2023). Copyright 2016 Elsevier, 2023 Elsevier.

The excellent mechanical strength, tunable biodegradation rate, and favorable biocompatibility make silk fibroin suitable candidates for bone regeneration, especially when silk fibroin is composited with other bioactive macromolecules or cells. For instance, porous silk-based scaffolds seeded with human MSCs have been widely utilized in bone tissue engineering (Meinel et al., 2004; Meinel et al., 2006). Besides, adipose-derived stromal cells (ADSCs), isolated from adipose tissue, also demonstrate multiple differentiation potential. Under osteogenic culture conditions, ADSCs can undergo osteogenic lineages with similar morphological and biological properties (Ribeiro et al., 2017). Importantly, it has been shown that ADSCs keep an abundant supply, because they can be harvested from lipoaspirate (Levi and Longaker, 2011). Therefore, ADSCs are a promising source for bone regeneration. Ribeiro et al. developed a porous 3D biotextile composed of weft-knitted silk fibroin (SF) yarns and polyethylene terephthalate (PET) monofilament which is seeded with ADSCs (Ribeiro et al., 2017). SF-PET scaffold showed expression of osteogenic-related markers, osteocalcin, and osteopontin, and represented strong extracellular matrix mineralization. A combination of osteogenic factors such as bone morphogenetic protein (BMP) and hydroxyapatite (HAp) can augment and encourage mineralization and bone integration ability (MacIntosh et al., 2008). It has been confirmed that BMP-2 can facilitate the recruitment and osteogenic differentiation of MSC (Kimura et al., 2010). HAp shows great chemical similarity and biological affinity to the natural bone inorganic matrix (Zhou and Lee, 2011). In the study of Shen et al., a cell-free silk fibroin (SF)/nano-hydroxyapatite (nHAp) scaffold was designed, loading BMP-2 containing SF microspheres and physically absorbed stromal cell-derived factor-1 (SDF-1). The main function of SDF-1 is to recruit MSCs to injury sites. The sequential release of SDF-1 and BMP-2 was achieved due to the burst release of SDF-1 and sustained release of BMP-2, which made the best use of these two molecules and ensured sequential migration and differentiation of MSC. *In vitro* study showed that this scaffold exhibited a significantly upregulated secret of calcium and osteocalcin than the concomitant released scaffold. Further, MSCs were labeled by D-Luciferin and the result showed the excellent cell migration and proliferation of this scaffold. After 12 weeks of implantation into the rat model with a critical size calvarial defect, the defect was completely bridged (Shen et al., 2016). Cells can sense their surroundings and translate them into cellular events. Recent studies suggest cell microenvironment plays an important role in bone repair (Guimaraes et al., 2022). Hu et al. (2023) designed and fabricated bone microenvironment–specific electroactive hydrogel using SF and MXene nanosheets (Figure 8A). SF bio-encapsulates MXene and inhibits the restacking and oxidation of MXene. This electroactive hydrogel promoted direct osteogenesis, leading to a re-established electrical microenvironment for efficient bone regeneration, and it also acts as a piezoresistive pressure transducer, which can potentially be utilized to monitor the electrophysiological microenvironment.

In addition to bone tissue engineering, silk-based material can be utilized to generate ameliorated orthopedic devices. In terms of unstable fractures, such as intraarticular fractures severe multiple fractures, open reduction, and internal fixation are common treatments in clinical. Attributed to the robustness, low price, and ease of accessibility, metals, such as titanium alloys and stainless steel, keep the most frequently used source of orthopedic devices. However, as foreign implants, metallic devices have a risk of infection, delayed healing, soft tissue irritation, and discomfort. Even worse, the invasive second surgical removal may cause severe complications (Zhang et al., 2012a). These limitations necessitate advanced absorbable screws which avoid surgical removal. Poly-L-lactic acid and polyglycolic acid have been utilized as absorbable materials, whereas their potential osteolysis effect, adverse degradation products, and incomplete bone remodeling hinder their translation (Eglin and Alini, 2008a; Li et al., 2016). Silk is a unique candidate for orthopedic devices due to its strong mechanical properties, tunable degradability, favorable biocompatibility, and permission to modify. After the proper treatment, silk-based material can be properly machined (Li et al., 2016) or remold (Guo et al., 2020) to meet the morphology and structure requirements of orthopedic devices. In order to preclude the negative impact of organic solvent, an aqueous and “green” process strategy inspired by the natural spinning process was developed to realize self-assembly. Li et al. (2016) fabricated a solid silk blank with excellent machinability (Figure 8B). The machined screw had a glossy surface and sharp edges. In terms of mechanical strength, the maximum bending modulus and structural stiffness were greatly improved compared to those solvent-based devices. On the other hand, the blending of dopants and delivery of bioactive compounds also became easier and more effective because of the mild process. Hence, this study not only successfully incorporated nano-hydroxyapatite to reduce the swelling ratio and enhanced mechanics in the hydrated state, but also functionalized with P24, a synthetic BMP2-related peptide, to promote osteoinductive effect. This research proved silks can be developed into molding and machinable biomaterials through a mild and green strategy, paving the way for the development of a new class of silk-based resorbable orthopedic devices.

### 5.2 Cartilage tissue engineering

Conventional clinic treatments for cartilage defects, such as arthroscopic debridement/lavage, microfracture/drilling, and ACI, are facing some inevitable limitations. To overcome these shortages, cartilage tissue engineering (CTE) has been proposed to seek new and effective treatments by optimizing conditions, such as the source of seed cells, scaffolds, and indispensable stimulating factors (Makris et al., 2015).

The seed cells used most frequently in CTE are the mature chondrocytes isolated from animal or human articular cartilage and MSCs. Chondrocytes obtained from articular cartilage tissues are generally regarded as an ideal candidate for seed cells, which exist only in cartilage and can directly secrete cartilage-specific matrix without external stimulus (Man et al., 2016). Chondrocytes can generate hyaline cartilage possessing similar characteristics to native cartilage through pellet culture (Zhao et al., 2020). Though tissue-engineered cartilage can be formed by culturing chondrocytes in a bioreactor (Zhao et al., 2016), the isolation of autologous chondrocytes is greatly restricted as it causes secondary injuries; on the other hand, the chondrocyte phenotype decreases and chondrogenic markers cease, which leads to insufficient cartilage repair (Zhao et al., 2020). Maintaining the primitive characteristics of high differentiation of chondrocytes thus becomes a fundamental target of CTE. MSCs with the capability of multidirectional differentiation potential to bones and chondrocytes, have been proposed to be loaded on silk fibroin-based hydrogels, providing many advantages as chondrocytes. For example, MSCs have extensive sources including bone marrow and adipose, and can be isolated more easily than chondrocytes (Nejadnik et al., 2010). Besides, MSCs have been proven to secret multiple paracrine factors such as interleukin-7, 8, 11(IL-7, 8, 11), enhancing cartilage regeneration and repair (Eltoukhy et al., 2018).

**FIGURE 9:**
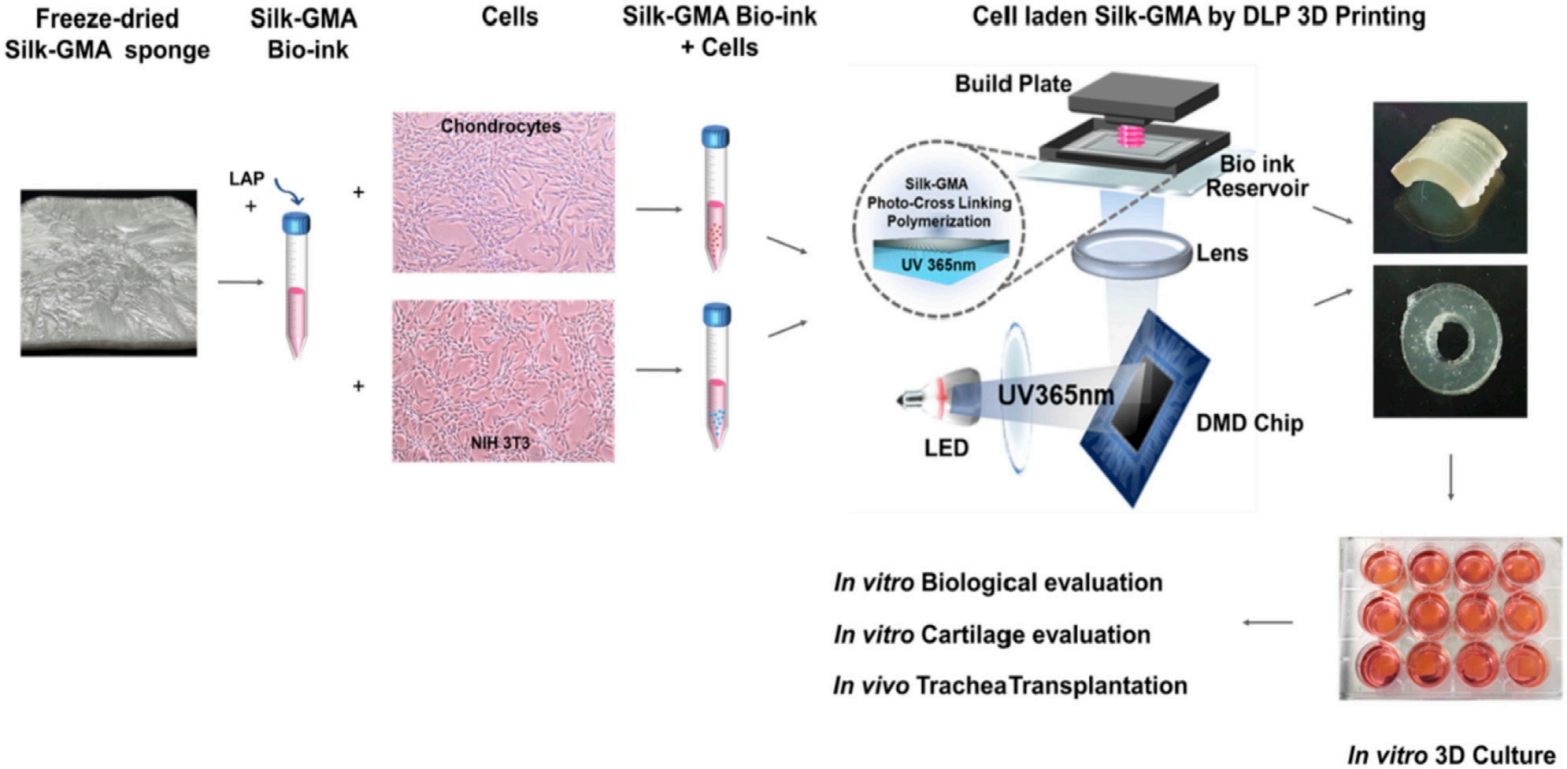
Chondrocyte-ladened silk-GMA bio-ink for DLP 3D printing technology has great potential in cartilage regeneration. Adapted with permission from ref (Hong et al., 2020) Copyright 2020 Elsevier.

Simultaneously, scaffolds play an equal role as seed cells in cartilage defect repair. Mechanical properties of scaffolds such as tensile strength and stiffness affect cellular functions significantly (Cheng et al., 2018); also, porous size and the three-dimensional structure of the scaffolds are pivotal elements in inducing, accelerating, and enhancing cell proliferation, differentiation, and ECM production of newly formed cartilage (Shi et al., 2017). Hydrogel scaffolds have shown great potential for encapsulating cells, growth factors, and other cell signaling factors for cartilage regeneration application. Agarose is a conventional and commonly accepted hydrogel that has been widely utilized for maintaining long-term chondrocyte cultures (Chao et al., 2010). However, the biomedical application of agarose hydrogels is restricted for agarose is immunogenic and nondegradable and the mechanical stiffness of agarose is difficult to modify (Chao et al., 2010). Therefore, SF materials, which show little to no inflammatory or immune response, have been fabricated into hydrogels for their tuned structural and mechanical properties, biocompatibility, and biodegradability. Digital light processing (DLP) is a promising 3D printing technology for scaffold fabrication in biomedical applications due to its high resolution, rapid printing efficiency, and capacity to maintain cell viability. Recently, Hong et al. fabricated silk fibroin and glycidyl-methacrylate (Silk-GMA) hydrogel with chondrocyte laded using 3D DLP printing for cartilage regeneration. Here the Silk-GMA act as bioink, providing powerful biocompatibility, tunable biodegradability, excellent mechanical stiffness to resist the physical stress during the DLP printing process, and various ways for crosslinking (Kim et al., 2018). The fabricated Silk-GMA sponge was loaded with cells of either chondrocyte or NIH3T3. And the photoinitiator, LAP, was added to induce a photopolymerization reaction when exposed to UV light. Integrating all evaluating results, the fabricated Silk-GMA showed excellent biocompatibility and remarkable biomechanical properties and provided great cartilage regeneration efficacy both *in vitro* and *in vivo* implantation (Figure 9) (Hong et al., 2020). This research proposed an approach for generating microenvironment-mimic scaffolds for cartilage regeneration using DLP and silk-based bio-inks, which is also promising for other tissue regeneration. Shi et al. designed a composite scaffold combining SF with gelatin using 3D printing technology to optimize the structure and function, giving the scaffold an appropriate mechanical stiffness and biodegradation rate balance for better chondrogenic differentiation. 3D printing was utilized to optimize the structure of the scaffold and silk fibroin along with gelatin acted as the structural matrix. Besides, BMSC affinity peptide E7 was added to the composite scaffold to functionally modify the BMSC homing ability. *In vitro*, BMSCs showed good growth on the SF-Gel-E7 scaffold, and the content of hydroxyproline (HYP) and glycosaminoglycan (GAG) increased statistically significantly, indicating a superior chondrocyte differentiation ability than the other scaffolds. *In vivo*, the SF-Gel group possessed better-formed neo-cartilage and showed superior performance in cartilage defect repairing, especially the SF-Gel-E7 group (Shi et al., 2017).

Besides, stimulating factors have been verified to be involved in supporting the formation of functional neo-cartilage and enhancing the quality of cartilage repair. Several growth factors are playing a regulating role in the homeostasis of mature functional cartilage, including Transforming Growth Factor-β (TGF-β), Fibroblast Growth Factors (FGFs), Insulin-Like Growth Factors (IGFs), Wingless (Wnt) Family, Notch Signaling Family and Hedgehog Family (Hh). For example, Hung et al. used water-based 3D printing materials polyurethane (PU) incorporating bioactive TGF-β3 to fabricate compliant scaffolds. These scaffolds induce MSCs to differentiate towards chondrocytes, thus accelerating the formation of cartilage and promoting cartilage repair (Hung et al., 2016). Tanshinone IIA (TAN), a traditional Chinese medicine extracted from Salviamiltiorrhiza Bunge (danshen), is easily redox, thus having a potential anti-inflammatory, anti-oxidant, and anti-apoptotic effect (Gao et al., 2008; Fan et al., 2009; Yin et al., 2012). In addition, it is previously verified that TAN can prevent cartilage degradation by inhibiting apoptosis and inflammatory factor expression levels (Jia et al., 2017), and TAN can significantly ameliorate rheumatoid arthritis (RA) by targeting neutrophils (Zhang et al., 2017). Based on these, Chen et al. designed and fabricate a novel TAN delivery silk fibroin scaffold to promote cartilage defect repairing, where TAN was used as a stimulating bioactive factor. TAN solution was mixed with SF solution followed by a certain ratio, and the expected SF-TAN scaffold was prepared by freeze-drying and self-assembled proteins. Then the efficacy was evaluated both *in vitro* and *in vivo*. After incubating with chondrocytes, the SF-TAN scaffold group showed hyaline-cartilage-like tissue and high content of cartilage-specific ECM. Furthermore, in the rabbit cartilage defect model, neo-cartilage was formed and showed perfect integration with surrounding tissues. And the newly formed cartilage possessed similar analogous biomedical mechanical properties and cartilage-specific ECM content. Hence, the TAN delivery silk fibroin scaffold can effectively and significantly promote cartilage defect repairing and thus can be potentially explored in clinical trials of cartilage defects.

### 5.3 Osteochondral interface tissue engineering

The natural osteochondral tissue widely existing in our joints contains both osteo and chondral components. The articular cartilage, subchondral bone, and the connected interface are the three main parts of osteochondral tissue. Besides, the connected interface is heterogeneous, gradient varying, and stable, which unifies osteo and chondral components as a single and complex whole (Nukavarapu and Dorcemus, 2013; Ribeiro et al., 2018). The defect or damage of osteochondral tissue can occur in any joint and impact all three parts. The osteochondral interface is particularly susceptible since there is no continuous collagen fiber. Even worse, this process is hard to reverse naturally because of its insufficient regenerative potential and complicated structure (Nukavarapu and Dorcemus, 2013). Hence, osteochondral tissue engineering is urged but confronts great challenges that the substitute should meet the structural and biological requirements of both articular cartilage and subchondral bone and ensure the compatibility and stability between these phases (Ribeiro et al., 2018). For this purpose, biphasic (Da et al., 2013), multiphasic (Levingstone et al., 2014) or stratified scaffolds have been developed. However, most of these are composited by inorganic phase and additional substrate, which leads to nonhomogeneity (Guo et al., 2017). To overcome this limitation, Guo et al. developed novel gradient silicified silk/R5 composites which are similar to the natural osteochondral interface. The R5 peptide is a bioinspired analog that can selectively promote the intermolecular interactions between biosilica particles and peptides. The R5/silk peptide solutions with different R5/silk molar ratios were separately added into a cylindrical container and gelled in sequence. Then, the biosilica particles were introduced and their concentration varied along the longitudinal R5/silk molar ratio, which resulted in gradient mechanical properties. Besides, the composites had varied pore sizes and can seed hMSCs. The *in vitro* study showed that silicified silk/R5 composites that encapsulated hMSCs had elevated calcium deposition and osteogenesis in comparison to unsilicified silk controls, indicating a promising approach in osteochondral tissue engineering (Guo et al., 2017).

## 6 Summary and outlook

Musculoskeletal degenerative diseases affect millions of individuals worldwide. To stimulate or augment the impaired or insufficient bone regeneration capacity, the current clinical practice includes distraction osteogenesis, bone substitutes, and bone-grafting methods, such as autologous and allogeneic grafts (Aronson, 1997; Giannoudis et al., 2005). However, the effect of osteogenesis keeps less than satisfactory. Similarly, the main strategies of osteoarthritis (OA) are conservative treatment, such as moderate exercise and pain management (Taruc-Uy and Lynch, 2013). There are rare disease-modifying drugs to alter or halt the pathological progression of OA (Karsdal et al., 2016). If conservative treatment fails, surgery, such as osteotomy and arthroplasty, is the only adoptable approach (Taruc-Uy and Lynch, 2013). Therefore, there is a growing consensus that novel treatments need to be developed and tissue engineering and regenerative medicine (TERM) suggest a promising solution (Bhattacharjee et al., 2017). TERM is an interdisciplinary field in which living cells, suitable biochemical and physio-chemical factors, and biocompatible materials are combined to ultimately repair or replace the damaged location (Berthiaume et al., 2011). TERM has been considered as a substitute to compensate for the limited supply of donor tissue and organ transplants. At the same time, the rejection reaction and mortality can be well-controlled (Nerem and Sambanis, 1995). Whilst challenges remain, advanced biomaterials, especially silk materials, and customized processing methods enable new frontiers for TERM.

Silk fibroin has emerged as a promising biomaterial for bone and cartilage tissue engineering due to its exceptional toughness, strength, biocompatibility, and versatility. Silk-based scaffolds are designed to mimic the *in-vivo* physiological microenvironment, regulating the fate of stem cells. To mimic the morphology of bone tissue, porous silk scaffolds are widely used, prepared by various processing methods such as freeze-drying, gas-foaming, and 3D printing. To obtain good biological outcomes, composite silk scaffolds are usually developed by incorporating osteogenic growth factors along with angiogenic growth factors or hydroxyapatite (HAp) in bone tissue engineering. Silk hydrogels are also good carriers for chondrogenic growth factors and some stimulative bioactive factors for maintaining the high differentiation characteristics of chondrocytes or guiding the differentiation potential of MSCs to chondrocytes. Additionally, BMSC affinity peptides and growth factor-related peptides were also grafted or encoded on the silks through some chemical methods or DNA recombinant technology for better functionality. Furthermore, the mechanical stiffness of silks has been well tunned. Silk-silk fiber composites are extensively prepared with enhanced mechanical properties to promote the development of osteogenesis. Besides providing an appropriate microenvironment for cells, silk is a rational choice for developing orthopedic devices due to its remarkable strength, toughness, ductility, biocompatibility, and biodegradability. Silk screws are promising resorbable fixation devices that address potential problems of osteolysis and adverse degradation product formation associated with metallic fixation devices.

Silk fibroin has high mechanical robustness, tunable biodegradation, and controlled biocompatibility, being attractive in biomedical applications especially musculoskeletal tissue repair and regeneration. Native and recombinant silks can be easily fabricated through aqueous-based bio-fabrication methods, including thermoplastic molding, crosslinking, and genetic engineering to integrate pre-designed cell niches. For improved regeneration efficiency in musculoskeletal tissue repair, a few new trends in silk material designs have emerged including designing soft and adaptable functional materials or enhancing mechanical properties to match the modules of native tissue, inducing gradient in materials for interfacial tissue regeneration, and genetic engineering protein sequence to incorporate cell niches. These functional designs of new silks aim to simulate multiple physical and biochemical cues of native bone and cartilage. For example, injectable silk protein hydrogels can fill and repair irregular shape defects, while mechanical enhancement and gradient stiffness silk protein hydrogels focused on the integrated repair of osteochondral defects. Induced mineralization in silk protein hydrogels was beneficial for mimicking the biochemical components of biological bones, promoting cellular differentiation. Growth factors were incorporated into the hydrogels to further regulate cell behavior while enhancing angiogenesis and osteogenesis. Overall, silk is one of the most capable biopolymers for tissue engineering with great translation potential. Up to now, there are already quite a few silk materials have been translated into FDA-approved biomedical devices, such as silk sutures (Surusil^®^, Suru; Sofsilk™, Covidien) and soft tissue scaffolds (Seri^®^ Surgical Scaffold, Allergan).

However, there are still some challenges in the design of silks in tissue engineering: 1) Despite many silk scaffolds emerging with a wide spectrum of formats including solutions, films, sponges, hydrogels, and particles, the only use of silks in clinical use is surgical mesh, sutures, and clothing which are all based on silk thread (Holland et al., 2018). Few silks were applied in clinical trials and commercialization of silks is still in the early stage. Most research on silks for bone and cartilage tissue engineering is still in the preclinical stage and has only developed as a model for small animal tests. The performance of these bioactive silks in humans cannot be predicted. 2) In *in vitro* experiments, silks are only a model for studying the osteogenic differentiation and chondrogenesis differentiation of MSCs considering their related gene expression and ECM deposits. Greater knowledge of 3D co-culture systems is required to generate dynamic tissues that can remodel like bone and cartilage. 3) Despite many efforts that have been done on the mechanical enhancement of silk-based scaffolds through a series of possessing methods, most of these regenerated silks still do not restore the excellent mechanical properties of native silks, due to the lack of suitable secondary and hierarchical structure. Tunning the secondary structures during the regeneration of silks and combining appropriate processing methods may offer the advantage of manipulating silk mechanical properties to the level of native silks in the future. 4) Diverse silk formats have been developed through a wide range of processing methods to meet the specific demand of particular tissue engineering. However, no silk protein-based formats can fully mimic the physical and biochemical cues of native musculoskeletal tissue. A “bottom-up” approach through DNA recombinant technology for rational silk design is promising. The structures and functions of silk-based materials can be tuned at the sequence level by incorporating non-natural amino acids, specific structural domains, and functionalized sequences.

In the future, efforts should be made to tailor silk properties for more precise tissue requirements. We expect further development and widespread utilization of high throughput material screening platforms to explore different material formulations, combinations of mechanical cues, biological niches, and growth factors, to improve regenerative outcomes. Moreover, we expect to see an expansion in the research to synergistically incorporate genetic engineering technology with machine learning and AI to rationally design and predict the properties of silk materials. With natural and recombinant silks receiving increasing attention from interdisciplinary researchers, further development of silk materials for musculoskeletal tissue engineering, taking them up to and beyond the stage of human trials, is hoped to be achieved in the near future through a cross-disciplinary coalition of material scientists, biomedical engineers, biologists, and medical doctors.

## References

[B1] Abdel-Sayed P. Pioletti D. P. (2015). Strategies for improving the repair of focal cartilage defects. Nanomedicine 10, 2893–2905. 10.2217/nnm.15.119 26377158

[B2] Addison W. N. Miller S. J. Ramaswamy J. Mansouri A. Kohn D. H. Mckee M. D. (2010). Phosphorylation-dependent mineral-type specificity for apatite-binding peptide sequences. Biomaterials 31, 9422–9430. 10.1016/j.biomaterials.2010.08.064 20943264 PMC2976791

[B3] Aigner T. B. Desimone E. Scheibel T. (2018). Biomedical applications of recombinant silk-based materials. Adv. Mater. 30, 1704636. 10.1002/adma.201704636 29436028

[B4] Altman G. H. Diaz F. Jakuba C. Calabro T. Horan R. L. Chen J. S. (2003). Silk-based biomaterials. Biomaterials 24, 401–416. 10.1016/s0142-9612(02)00353-8 12423595

[B5] An B. Tang-Schomer M. D. Huang W. W. He J. Y. Jones J. A. Lewis R. V. (2015). Physical and biological regulation of neuron regenerative growth and network formation on recombinant dragline silks. Biomaterials 48, 137–146. 10.1016/j.biomaterials.2015.01.044 25701039 PMC4353650

[B6] Aronson J. (1997). Current concepts review - limb-lengthening, skeletal reconstruction, and bone transport with the ilizarov method*. J. Bone Jt. Surg. Am. 79, 1243–1258. 10.2106/00004623-199708000-00019 9278087

[B7] Bakhshandeh B. Nateghi S. S. Gazani M. M. Dehghani Z. Mohammadzadeh F. (2021). A review on advances in the applications of spider silk in biomedical issues. Int. J. Biol. Macromol. 192, 258–271. 10.1016/j.ijbiomac.2021.09.201 34627845

[B8] Barth H. D. Zimmermann E. A. Schaible E. Tang S. Y. Alliston T. Ritchie R. O. (2011). Characterization of the effects of x-ray irradiation on the hierarchical structure and mechanical properties of human cortical bone. Biomaterials 32, 8892–8904. 10.1016/j.biomaterials.2011.08.013 21885114 PMC4405888

[B9] Begum R. Perriman A. W. Su B. Scarpa F. Kafienah W. (2020). Chondroinduction of mesenchymal stem cells on cellulose-silk composite nanofibrous substrates: The role of substrate elasticity. Front. Bioeng. Biotechnol. 8, 197. 10.3389/fbioe.2020.00197 32266231 PMC7096586

[B10] Berthiaume F. Maguire T. J. Yarmush M. L. (2011). Tissue engineering and regenerative medicine: History, progress, and challenges. Annu. Rev. Chem. Biomol. Eng. 2, 403–430. 10.1146/annurev-chembioeng-061010-114257 22432625

[B11] Bhattacharjee P. Kundu B. Naskar D. Kim H. W. Maiti T. K. Bhattacharya D. (2017). Silk scaffolds in bone tissue engineering: An overview. Acta Biomater. 63, 1–17. 10.1016/j.actbio.2017.09.027 28941652

[B12] Bini E. Foo C. W. P. Huang J. Karageorgiou V. Kitchel B. Kaplan D. L. (2006). RGD-functionalized bioengineered spider dragline silk biomaterial. Biomacromolecules 7, 3139–3145. 10.1021/bm0607877 17096543

[B13] Briggs A. M. Cross M. J. Hoy D. G. Sanchez-Riera L. Blyth F. M. Woolf A. D. (2016). Musculoskeletal health conditions represent a global threat to healthy aging: A report for the 2015 world health organization world report on ageing and health. Gerontologist 56, S243–S255. 10.1093/geront/gnw002 26994264

[B14] Broaddus W. C. Holloway K. L. Winters C. J. Bullock M. R. Graham R. S. Mathern B. E. (2002). Titanium miniplates or stainless steel wire for cranial fixation: A prospective randomized comparison. J. Neurosurg. 96, 244–247. 10.3171/jns.2002.96.2.0244 11838797

[B15] Brodsky B. Persikov A. V. (2005). Molecular structure of the collagen triple helix. Adv. Protein Chem. 70, 301–339. 10.1016/S0065-3233(05)70009-7 15837519

[B16] Chao P. H. Yodmuang S. Wang X. Sun L. Kaplan D. L. Vunjak-Novakovic G. (2010). Silk hydrogel for cartilage tissue engineering. J. Biomed. Mater Res. B Appl. Biomater. 95, 84–90. 10.1002/jbm.b.31686 20725950 PMC3079331

[B17] Chen W. Xu Y. Li H. Dai Y. Zhou G. Zhou Z. (2020). Tanshinone IIA delivery silk fibroin scaffolds significantly enhance articular cartilage defect repairing via promoting cartilage regeneration. ACS Appl. Mater Interfaces 12, 21470–21480. 10.1021/acsami.0c03822 32314911

[B18] Cheng G. Davoudi Z. Xing X. Yu X. Cheng X. Li Z. (2018). Advanced silk fibroin biomaterials for cartilage regeneration. ACS Biomaterials Sci. Eng. 4, 2704–2715. 10.1021/acsbiomaterials.8b00150 33434996

[B19] Cheng W. Ding Z. Zheng X. Lu Q. Kong X. Zhou X. (2020). Injectable hydrogel systems with multiple biophysical and biochemical cues for bone regeneration. Biomaterials Sci. 8, 2537–2548. 10.1039/d0bm00104j PMC7204512 32215404

[B20] Clarke B. (2008). Normal bone anatomy and physiology. Clin. J. Am. Soc. Nephrol. 3 (3), S131–S139. 10.2215/cjn.04151206 18988698 PMC3152283

[B21] Da H. Jia S. J. Meng G. L. Cheng J. H. Zhou W. Xiong Z. (2013). The impact of compact layer in biphasic scaffold on osteochondral tissue engineering. PLoS One 8, e54838. 10.1371/journal.pone.0054838 23382984 PMC3557302

[B22] Deng C. Yang J. He H. Ma Z. Wang W. Zhang Y. (2021). 3D bio-printed biphasic scaffolds with dual modification of silk fibroin for the integrated repair of osteochondral defects. Biomaterials Sci. 9, 4891–4903. 10.1039/d1bm00535a 34047307

[B23] Dimitriou R. Jones E. Mcgonagle D. Giannoudis P. V. (2011). Bone regeneration: Current concepts and future directions. BMC Med. 9, 66. 10.1186/1741-7015-9-66 21627784 PMC3123714

[B24] Ding X. Zhu M. Xu B. Zhang J. Zhao Y. Ji S. (2014). Integrated trilayered silk fibroin scaffold for osteochondral differentiation of adipose-derived stem cells. Acs Appl. Mater. Interfaces 6, 16696–16705. 10.1021/am5036708 25210952

[B25] Dinjaski N. Huang W. Kaplan D. L. (2018). “Recursive directional ligation approach for cloning recombinant spider silks,” in Peptide self-assembly: Methods and protocols. Editors NILSSON B. L. DORAN T. M. 10.1007/978-1-4939-7811-3_10 29744835

[B26] Dinjaski N. Plowright R. Zhou S. Belton D. J. Perry C. C. Kaplan D. L. (2017). Osteoinductive recombinant silk fusion proteins for bone regeneration. Acta Biomater. 49, 127–139. 10.1016/j.actbio.2016.12.002 27940162 PMC5253115

[B27] Docheva D. Muller S. A. Majewski M. Evans C. H. (2015). Biologics for tendon repair. Adv. Drug Deliv. Rev. 84, 222–239. 10.1016/j.addr.2014.11.015 25446135 PMC4519231

[B28] Eglin D. Alini M. (2008a). Degradable polymeric materials for osteosynthesis: Tutorial. Eur. Cell Mater 16, 80–91. 10.22203/ecm.v016a09 19101891

[B29] Eglin D. Alini M. (2008b). Degradable polymeric materials for osteosynthesis: Tutorial. Eur. Cells Mater. 16, 80–91. 10.22203/ecm.v016a09 19101891

[B30] Einhorn T. A. (1998). The cell and molecular biology of fracture healing. Clin. Orthop. Relat. Res. S7-21, S7–S21. 10.1097/00003086-199810001-00003 9917622

[B31] Eltoukhy H. S. Sinha G. Moore C. A. Gergues M. Rameshwar P. (2018). Secretome within the bone marrow microenvironment: A basis for mesenchymal stem cell treatment and role in cancer dormancy. Biochimie 155, 92–103. 10.1016/j.biochi.2018.05.018 29859990

[B32] Emans P. J. Van Rhijn L. W. Welting T. J. M. Cremers A. Wijnands N. Spaapen F. (2010). Autologous engineering of cartilage. Proc. Natl. Acad. Sci. U. S. A. 107, 3418–3423. 10.1073/pnas.0907774107 20133690 PMC2840469

[B33] Endisha H. Rockel J. Jurisica I. Kapoor M. (2018). The complex landscape of microRNAs in articular cartilage: Biology, pathology, and therapeutic targets. JCI Insight 3, e121630. 10.1172/jci.insight.121630 30185670 PMC6171796

[B34] Fan G.-W. Gao X.-M. Wang H. Zhu Y. Zhang J. Hu L.-M. (2009). The anti-inflammatory activities of Tanshinone IIA, an active component of TCM, are mediated by estrogen receptor activation and inhibition of iNOS. J. Steroid Biochem. Mol. Biol. 113, 275–280. 10.1016/j.jsbmb.2009.01.011 19429433

[B35] Farah S. Anderson D. G. Langer R. (2016). Physical and mechanical properties of PLA, and their functions in widespread applications - a comprehensive review. Adv. Drug Deliv. Rev. 107, 367–392. 10.1016/j.addr.2016.06.012 27356150

[B36] Farokhi M. Mottaghitalab F. Samani S. Shokrgozar M. A. Kundu S. C. Reis R. L. (2018). Silk fibroin/hydroxyapatite composites for bone tissue engineering. Biotechnol. Adv. 36, 68–91. 10.1016/j.biotechadv.2017.10.001 28993220

[B37] Florencio-Silva R. Sasso G. R. Sasso-Cerri E. Simoes M. J. Cerri P. S. (2015). Biology of bone tissue: Structure, function, and factors that influence bone cells. Biomed. Res. Int. 2015, 1–17. 10.1155/2015/421746 PMC4515490 26247020

[B38] Funda G. Taschieri S. Bruno G. A. Grecchi E. Paolo S. Girolamo D. (2020), Nanotechnology scaffolds for alveolar bone regeneration. Materials (Basel) 13 10.3390/ma13010201 PMC6982209 31947750

[B39] Gao M. Yang G. Q. Pi R. B. Li R. F. Wang P. Zhang H. J. (2008). Tanshinone IIA protects neonatal rat cardiomyocytes from adriamycin-induced apoptosis. Transl. Res. 151, 79–87. 10.1016/j.trsl.2007.11.005 18201675

[B40] Giannoudis P. V. Dinopoulos H. Tsiridis E. (2005). Bone substitutes: An update. Injury 36 (3), S20–S27. 10.1016/j.injury.2005.07.029 16188545

[B41] Giesa T. Arslan M. Pugno N. M. Buehler M. J. (2011). Nanoconfinement of spider silk fibrils begets superior strength, extensibility, and toughness. Nano Lett. 11, 5038–5046. 10.1021/nl203108t 21967633

[B42] Gosline J. M. Guerette P. A. Ortlepp C. S. Savage K. N. (1999). The mechanical design of spider silks: From fibroin sequence to mechanical function. J. Exp. Biol. 202, 3295–3303. 10.1242/jeb.202.23.3295 10562512

[B43] Grogan S. P. Chung P. H. Soman P. Chen P. Lotz M. K. Chen S. (2013). Digital micromirror device projection printing system for meniscus tissue engineering. Acta Biomater. 9, 7218–7226. 10.1016/j.actbio.2013.03.020 23523536 PMC3685281

[B44] Guimaraes C. F. Marques A. P. Reis R. L. (2022). Pushing the natural frontier: Progress on the integration of biomaterial cues toward combinatorial biofabrication and tissue engineering. Adv. Mater. 34, 2105645. 10.1002/adma.202105645 35419887

[B45] Guo C. Li C. Vu H. V. Hanna P. Lechtig A. Qiu Y. (2020). Thermoplastic moulding of regenerated silk. Nat. Mater 19, 102–108. 10.1038/s41563-019-0560-8 31844276 PMC6986341

[B46] Guo J. Li C. Ling S. Huang W. Chen Y. Kaplan D. L. (2017). Multiscale design and synthesis of biomimetic gradient protein/biosilica composites for interfacial tissue engineering. Biomaterials 145, 44–55. 10.1016/j.biomaterials.2017.08.025 28843732 PMC5610098

[B47] Hung K. C. Tseng C. S. Dai L. G. Hsu S. H. (2016). Water-based polyurethane 3D printed scaffolds with controlled release function for customized cartilage tissue engineering. Biomaterials 83, 156–168. 10.1016/j.biomaterials.2016.01.019 26774563

[B48] Hasturk O. Jordan K. E. Choi J. Kaplan D. L. (2020). Enzymatically crosslinked silk and silk-gelatin hydrogels with tunable gelation kinetics, mechanical properties and bioactivity for cell culture and encapsulation. Biomaterials 232, 119720. 10.1016/j.biomaterials.2019.119720 31896515 PMC7667870

[B49] Hedhammar M. Rising A. Grip S. Martinez A. S. Nordling K. Casals C. (2008). Structural properties of recombinant nonrepetitive and repetitive parts of major ampullate spidroin 1 from Euprosthenops australis: Implications for fiber formation. Biochemistry 47, 3407–3417. 10.1021/bi702432y 18293938

[B50] Holland C. Numata K. Rnjak-Kovacina J. Seib F. P. (2018). The biomedical use of silk: Past, present, future. Adv. Healthc. Mater. 8, 1800465. 10.1002/adhm.201800465 30238637

[B51] Hong H. Seo Y. B. Kim D. Y. Lee J. S. Lee Y. J. Lee H. (2020). Digital light processing 3D printed silk fibroin hydrogel for cartilage tissue engineering. Biomaterials 232, 119679. 10.1016/j.biomaterials.2019.119679 31865191

[B52] Hu Z. C. Lu J. Q. Zhang T. W. Liang H. F. Yuan H. Su D. H. (2023). Piezoresistive MXene/Silk fibroin nanocomposite hydrogel for accelerating bone regeneration by Re-establishing electrical microenvironment. Bioact. Mater. 22, 1–17. 10.1016/j.bioactmat.2022.08.025 36203961 PMC9513113

[B53] Huang W. Ling S. Li C. Omenetto F. G. Kaplan D. L. (2018). Silkworm silk-based materials and devices generated using bio-nanotechnology. Chem. Soc. Rev. 47, 6486–6504. 10.1039/c8cs00187a 29938722 PMC6113080

[B54] Huang W. W. Ebrahimi D. Dinjaski N. Tarakanova A. Buehler M. J. Wong J. Y. (2017). Synergistic integration of experimental and simulation approaches for the de Novo design of silk-based materials. Accounts Chem. Res. 50, 866–876. 10.1021/acs.accounts.6b00616 PMC9310429 28191922

[B55] Huang W. W. Krishnaji S. Hu X. Kaplan D. Cebe P. (2011). Heat capacity of spider silk-like block copolymers. Macromolecules 44, 5299–5309. 10.1021/ma200563t 23869111 PMC3712525

[B56] Hutmacher D. W. (2000). Scaffolds in tissue engineering bone and cartilage. Biomaterials 21, 2529–2543. 10.1016/s0142-9612(00)00121-6 11071603

[B57] Imola M. J. Hamlar D. D. Shao W. Chowdhury K. Tatum S. (2001). Resorbable plate fixation in pediatric craniofacial surgery: Long-term outcome. Archives facial plastic Surg. 3, 79–90. 10.1001/archfaci.3.2.79 11368657

[B58] Jia P.-T. Zhang X.-L. Zuo H.-N. Lu X. Li L. (2017). Articular cartilage degradation is prevented by tanshinone IIA through inhibiting apoptosis and the expression of inflammatory cytokines. Mol. Med. Rep. 16, 6285–6289. 10.3892/mmr.2017.7340 28849083

[B59] Jin Y. Kundu B. Cai Y. Kundu S. C. Yao J. (2015). Bio-inspired mineralization of hydroxyapatite in 3D silk fibroin hydrogel for bone tissue engineering. Colloids Surfaces B-Biointerfaces 134, 339–345. 10.1016/j.colsurfb.2015.07.015 26209967

[B60] Karsdal M. A. Michaelis M. Ladel C. Siebuhr A. S. Bihlet A. R. Andersen J. R. (2016). Disease-modifying treatments for osteoarthritis (DMOADs) of the knee and hip: Lessons learned from failures and opportunities for the future. Osteoarthr. Cartil. 24, 2013–2021. 10.1016/j.joca.2016.07.017 27492463

[B61] Keten S. Xu Z. Ihle B. Buehler M. J. (2010). Nanoconfinement controls stiffness, strength and mechanical toughness of beta-sheet crystals in silk. Nat. Mater. 9, 359–367. 10.1038/nmat2704 20228820

[B62] Khader B. A. Towler M. R. (2016). Materials and techniques used in cranioplasty fixation: A review. Mater. Sci. Eng. C-Materials Biol. Appl. 66, 315–322. 10.1016/j.msec.2016.04.101 27207068

[B63] Khorshidi S. Karimi-Soflou R. Karkhaneh A. (2021). A hydrogel/particle composite with a gradient of oxygen releasing microparticle for concurrent osteogenic and chondrogenic differentiation in a single scaffold. Colloids Surfaces B-Biointerfaces, 207, 112007, 10.1016/j.colsurfb.2021.112007 34339972

[B64] Kim S. H. Yeon Y. K. Lee J. M. Chao J. R. Lee Y. J. Seo Y. B. (2018). Precisely printable and biocompatible silk fibroin bioink for digital light processing 3D printing. Nat. Commun. 9, 1620. 10.1038/s41467-018-03759-y 29693652 PMC5915392

[B65] Kim U. J. Park J. Kim H. J. Wada M. Kaplan D. L. (2005). Three-dimensional aqueous-derived biomaterial scaffolds from silk fibroin. Biomaterials 26, 2775–2785. 10.1016/j.biomaterials.2004.07.044 15585282

[B66] Kimura Y. Miyazaki N. Hayashi N. Otsuru S. Tamai K. Kaneda Y. (2010). Controlled release of bone morphogenetic protein-2 enhances recruitment of osteogenic progenitor cells for de novo generation of bone tissue. Tissue Eng. Part A 16, 1263–1270. 10.1089/ten.tea.2009.0322 19886805

[B67] Krishnakumar G. S. Sampath S. Muthusamy S. John M. A. (2019). Importance of crosslinking strategies in designing smart biomaterials for bone tissue engineering: A systematic review. Mater Sci. Eng. C Mater Biol. Appl. 96, 941–954. 10.1016/j.msec.2018.11.081 30606606

[B68] Krishnan Y. Grodzinsky A. J. (2018). Cartilage diseases. Matrix Biol. 71-72, 51–69. 10.1016/j.matbio.2018.05.005 29803938 PMC6146013

[B69] Kundu B. Bano S. Patra C. Engel F. B. Yadavalli V. K. Kundu S. C. (2014). Silk proteins for biomedical applications: Bioengineering perspectives. Prog. Polym. Sci. 39, 251–267. 10.1016/j.progpolymsci.2013.09.002

[B70] Kundu B. Rajkhowa R. Kundu S. C. Wang X. (2013). Silk fibroin biomaterials for tissue regenerations. Adv. Drug Deliv. Rev. 65, 457–470. 10.1016/j.addr.2012.09.043 23137786

[B71] Levi B. Longaker M. T. (2011). Concise review: Adipose-derived stromal cells for skeletal regenerative medicine. Stem Cells 29, 576–582. 10.1002/stem.612 21305671 PMC3323288

[B72] Levingstone T. J. Matsiko A. Dickson G. R. O'Brien F. J. Gleeson J. P. (2014). A biomimetic multi-layered collagen-based scaffold for osteochondral repair. Acta Biomater. 10, 1996–2004. 10.1016/j.actbio.2014.01.005 24418437

[B73] Li C. Hotz B. Ling S. Guo J. Haas D. S. Marelli B. (2016). Regenerated silk materials for functionalized silk orthopedic devices by mimicking natural processing. Biomaterials 110, 24–33. 10.1016/j.biomaterials.2016.09.014 27697669 PMC5104183

[B74] Lin H. Zhang D. Alexander P. G. Yang G. Tan J. Cheng A. W.-M. (2013). Application of visible light-based projection stereolithography for live cell-scaffold fabrication with designed architecture. Biomaterials 34, 331–339. 10.1016/j.biomaterials.2012.09.048 23092861 PMC3612429

[B75] Lin S. C. Ryu S. Tokareva O. Gronau G. Jacobsen M. M. Huang W. W. (2015). Predictive modelling-based design and experiments for synthesis and spinning of bioinspired silk fibres. Nat. Commun. 6, 6892. 10.1038/ncomms7892 26017575 PMC4996357

[B76] Liu K. Shi Z. Zhang S. Zhou Z. Sun L. Xu T. (2018). A silk cranial fixation system for neurosurgery. Adv. Healthc. Mater 7, e1701359. 10.1002/adhm.201701359 29377631

[B77] Lv Z. Hu T. Bian Y. Wang G. Wu Z. Li H. (2022). A MgFe-LDH nanosheet-incorporated smart thermo-responsive hydrogel with controllable growth factor releasing capability for bone regeneration. Advanced materials 10.1002/adma.202206545 36426823

[B78] Ma D. Wang Y. Dai W. (2018). Silk fibroin-based biomaterials for musculoskeletal tissue engineering. Mater. Sci. Eng. C 89, 456–469. 10.1016/j.msec.2018.04.062 29752118

[B79] Macintosh A. C. Kearns V. R. Crawford A. Hatton P. V. (2008). Skeletal tissue engineering using silk biomaterials. J. Tissue Eng. Regen. Med. 2, 71–80. 10.1002/term.68 18383453

[B80] Majidinia M. Sadeghpour A. Yousefi B. (2018). The roles of signaling pathways in bone repair and regeneration. J. Cell. Physiology 233, 2937–2948. 10.1002/jcp.26042 28590066

[B81] Makris E. A. Gomoll A. H. Malizos K. N. Hu J. C. Athanasiou K. A. (2015). Repair and tissue engineering techniques for articular cartilage. Nat. Rev. Rheumatol. 11, 21–34. 10.1038/nrrheum.2014.157 25247412 PMC4629810

[B82] Man Z. Hu X. Liu Z. Huang H. Meng Q. Zhang X. (2016). Transplantation of allogenic chondrocytes with chitosan hydrogel-demineralized bone matrix hybrid scaffold to repair rabbit cartilage injury. Biomaterials 108, 157–167. 10.1016/j.biomaterials.2016.09.002 27636153

[B83] Mansfield J. C. Bell J. S. Winlove C. P. (2015). The micromechanics of the superficial zone of articular cartilage. Osteoarthr. Cartil. 23, 1806–1816. 10.1016/j.joca.2015.05.030 26050867

[B84] Marx R. E. (2007)., 19. v, 455–466. 10.1016/j.coms.2007.07.008 Bone and bone graft healing Oral Maxillofac. Surg. Clin. North Am. 18088897

[B85] Meinel L. Betz O. Fajardo R. Hofmann S. Nazarian A. Cory E. (2006). Silk based biomaterials to heal critical sized femur defects. Bone 39, 922–931. 10.1016/j.bone.2006.04.019 16757219

[B86] Meinel L. Karageorgiou V. Hofmann S. Fajardo R. Snyder B. Li C. (2004). Engineering bone-like tissue *in vitro* using human bone marrow stem cells and silk scaffolds. J. Biomed. Mater Res. A 71, 25–34. 10.1002/jbm.a.30117 15316936

[B87] Mirahmadi F. Tafazzoli-Shadpour M. Shokrgozar M. A. Bonakdar S. (2013). Enhanced mechanical properties of thermosensitive chitosan hydrogel by silk fibers for cartilage tissue engineering. Mater. Sci. Eng. C-Materials Biol. Appl. 33, 4786–4794. 10.1016/j.msec.2013.07.043 24094188

[B88] Murphy A. R. Kaplan D. L. (2009). Biomedical applications of chemically-modified silk fibroin. J. Mater. Chem. 19, 6443–6450. 10.1039/b905802h 20161439 PMC2790051

[B89] Naba A. Clauser K. R. Ding H. Whittaker C. A. Carr S. A. Hynes R. O. (2016). The extracellular matrix: Tools and insights for the "omics" era. Matrix Biol. 49, 10–24. 10.1016/j.matbio.2015.06.003 26163349 PMC5013529

[B90] Naskar D. Ghosh A. K. Mandal M. Das P. Nandi S. K. Kundu S. C. (2017). Dual growth factor loaded nonmulberry silk fibroin/carbon nanofiber composite 3D scaffolds for *in vitro* and *in vivo* bone regeneration. Biomaterials 136, 67–85. 10.1016/j.biomaterials.2017.05.014 28521202

[B91] Nejadnik H. Hui J. H. Choong E. P. F. Tai B.-C. Lee E. H. (2010). Autologous bone marrow-derived mesenchymal stem cells versus autologous chondrocyte implantation an observational cohort study. Am. J. Sports Med. 38, 1110–1116. 10.1177/0363546509359067 20392971

[B92] Nerem R. M. Sambanis A. (1995). Tissue engineering: From biology to biological substitutes. Tissue Eng. 1, 3–13. 10.1089/ten.1995.1.3 19877911

[B93] Neubauer V. J. Scheibel T. (2020). Spider silk fusion proteins for controlled collagen binding and biomineralization. Acs Biomaterials Sci. Eng. 6, 5599–5608. 10.1021/acsbiomaterials.0c00818 33320578

[B94] Nukavarapu S. P. Dorcemus D. L. (2013). Osteochondral tissue engineering: Current strategies and challenges. Biotechnol. Adv. 31, 706–721. 10.1016/j.biotechadv.2012.11.004 23174560

[B95] Panilaitis B. Altman G. H. Chen J. S. Jin H. J. Karageorgiou V. Kaplan D. L. (2003). Macrophage responses to silk. Biomaterials 24, 3079–3085. 10.1016/s0142-9612(03)00158-3 12895580

[B96] Pei Y. Wang L. Tang K. Kaplan D. L. (2021). Biopolymer nanoscale assemblies as building blocks for new materials: A review. Adv. Funct. Mater. 31, 2008552. 10.1002/adfm.202008552

[B97] Perez-Rigueiro J. Elices M. Llorca J. Viney C. (2002). Effect of degumming on the tensile properties of silkworm (*Bombyx mori*) silk fiber. J. Appl. Polym. Sci. 84, 1431–1437. 10.1002/app.10366

[B98] Perrone G. S. Leisk G. G. Lo T. J. Moreau J. E. Haas D. S. Papenburg B. J. (2014). The use of silk-based devices for fracture fixation. Nat. Commun. 5, 3385. 10.1038/ncomms4385 24594992

[B99] Ribeiro V. P. Pina S. Oliveira J. M. Reis R. L. (2018). Silk fibroin-based hydrogels and scaffolds for osteochondral repair and regeneration. Adv. Exp. Med. Biol., 1058, 305–325. 10.1007/978-3-319-76711-6_14 29691828

[B100] Ribeiro V. P. Silva-Correia J. Nascimento A. I. Da Silva Morais A. Marques A. P. Ribeiro A. S. (2017). Silk-based anisotropical 3D biotextiles for bone regeneration. Biomaterials 123, 92–106. 10.1016/j.biomaterials.2017.01.027 28161684

[B101] Rockwood D. N. Preda R. C. Yucel T. Wang X. Lovett M. L. Kaplan D. L. (2011). Materials fabrication from *Bombyx mori* silk fibroin. Nat. Protoc. 6, 1612–1631. 10.1038/nprot.2011.379 21959241 PMC3808976

[B102] Shao J. Zhang W. Yang T. (2015). Using mesenchymal stem cells as a therapy for bone regeneration and repairing. Biol. Res. 48, 62. 10.1186/s40659-015-0053-4 26530042 PMC4630918

[B103] Shen X. Zhang Y. Gu Y. Xu Y. Liu Y. Li B. (2016). Sequential and sustained release of SDF-1 and BMP-2 from silk fibroin-nanohydroxyapatite scaffold for the enhancement of bone regeneration. Biomaterials 106, 205–216. 10.1016/j.biomaterials.2016.08.023 27566869

[B104] Shi W. Sun M. Hu X. Ren B. Cheng J. Li C. (2017). Structurally and functionally optimized silk-fibroin-gelatin scaffold using 3D printing to repair cartilage injury *in vitro* and *in vivo* . Adv. Mater 29, 1701089. 10.1002/adma.201701089 28585319

[B105] Sofia S. Mccarthy M. B. Gronowicz G. Kaplan D. L. (2001). Functionalized silk-based biomaterials for bone formation. J. Biomed. Mater. Res. 54, 139–148. 10.1002/1097-4636(200101)54:1<139::aid-jbm17>3.0.co;2-7 11077413

[B106] Sponner A. Vater W. Monajembashi S. Unger E. Grosse F. Weisshart K. (2007). Composition and hierarchical organisation of a spider silk. Plos One 2, e998. 10.1371/journal.pone.0000998 17912375 PMC1994588

[B107] Sui L. Wang M. Han Q. Yu L. Zhang L. Zheng L. (2018). A novel Lipidoid-MicroRNA formulation promotes calvarial bone regeneration. Biomaterials 177, 88–97. 10.1016/j.biomaterials.2018.05.038 29886386 PMC6019203

[B108] Taruc-Uy R. L. Lynch S. A. (2013). Diagnosis and treatment of osteoarthritis. Prim. Care, 40(4), 821–836. 10.1016/j.pop.2013.08.003 24209720

[B109] Thakur G. Rodrigues F. C. Singh K. (2018). Crosslinking biopolymers for advanced drug delivery and tissue engineering applications. Adv. Exp. Med. Biol. 1078, 213–231. 10.1007/978-981-13-0950-2_11 30357625

[B110] Umlauf D. Frank S. Pap T. Bertrand J. (2010). Cartilage biology, pathology, and repair. Cell Mol. Life Sci. 67, 4197–4211. 10.1007/s00018-010-0498-0 20734104 PMC11115553

[B111] Vepari C. Kaplan D. L. (2007). Silk as a biomaterial. Prog. Polym. Sci. 32, 991–1007. 10.1016/j.progpolymsci.2007.05.013 19543442 PMC2699289

[B112] Vinatier C. Mrugala D. Jorgensen C. Guicheux J. Noel D. (2009). Cartilage engineering: A crucial combination of cells, biomaterials and biofactors. Trends Biotechnol. 27, 307–314. 10.1016/j.tibtech.2009.02.005 19329205

[B113] Wang H. Leeuwenburgh S. C. Li Y. Jansen J. A. (2012). The use of micro- and nanospheres as functional components for bone tissue regeneration. Tissue Eng. Part B Rev. 18, 24–39. 10.1089/ten.teb.2011.0184 21806489 PMC3262980

[B114] Wang Y. Z. Kim U. J. Blasioli D. J. Kim H. J. Kaplan D. L. (2005). *In vitro* cartilage tissue engineering with 3D porous aqueous-derived silk scaffolds and mesenchymal stem cells. Biomaterials 26, 7082–7094. 10.1016/j.biomaterials.2005.05.022 15985292

[B115] Wei W. Ma Y. Z. Yao X. D. Zhou W. Y. Wang X. Z. Li C. L. (2021). Advanced hydrogels for the repair of cartilage defects and regeneration. Bioact. Mater. 6, 998–1011. 10.1016/j.bioactmat.2020.09.030 33102942 PMC7557878

[B116] Włodarczyk-Biegun M. K. Del Campo A. (2017). 3D bioprinting of structural proteins. Biomaterials 134, 180–201. 10.1016/j.biomaterials.2017.04.019 28477541

[B117] Wu J. Zheng K. Huang X. Liu J. Liu H. Boccaccini A. R. (2019). Thermally triggered injectable chitosan/silk fibroin/bioactive glass nanoparticle hydrogels for *in-situ* bone formation in rat calvarial bone defects. Acta Biomater. 91, 60–71. 10.1016/j.actbio.2019.04.023 30986530

[B118] Wu S. L. Liu X. M. Yeung K. W. K. Liu C. S. Yang X. J. (2014). Biomimetic porous scaffolds for bone tissue engineering. Mater. Sci. Eng. R-Reports 80, 1–36. 10.1016/j.mser.2014.04.001

[B119] Xu G. Gong L. Yang Z. Liu X. Y. (2014). What makes spider silk fibers so strong? From molecular-crystallite network to hierarchical network structures. Soft Matter 10, 2116–2123. 10.1039/c3sm52845f 24652059

[B120] Xu G. Ding Z. Lu Q. Zhang X. Zhou X. Xiao L. (2020). Electric field-driven building blocks for introducing multiple gradients to hydrogels. Protein & Cell 11, 267–285. 10.1007/s13238-020-00692-z 32048173 PMC7093350

[B121] Yang Y. Yao X. Li X. Guo C. Li C. Liu L. (2021). Non-mulberry silk fiber-based scaffolds reinforced by PLLA porous microspheres for auricular cartilage: An *in vitro* study. Int. J. Biol. Macromol. 182, 1704–1712. 10.1016/j.ijbiomac.2021.05.145 34052269

[B122] Yin X. Yin Y. Cao F.-L. Chen Y.-F. Peng Y. Hou W.-G. (2012). Tanshinone IIA attenuates the inflammatory response and apoptosis after traumatic injury of the spinal cord in adult rats. Plos One 7, e38381. 10.1371/journal.pone.0038381 22675554 PMC3365897

[B123] Yodmuang S. Mcnamara S. L. Nover A. B. Mandal B. B. Aganwal M. Kelly T.-A. N. (2015). Silk microfiber-reinforced silk hydrogel composites for functional cartilage tissue repair. Acta Biomater. 11, 27–36. 10.1016/j.actbio.2014.09.032 25281788 PMC4256092

[B124] Zhang W. Zhang Y. Zhang A. Ling C. Sheng R. Li X. (2021). Enzymatically crosslinked silk-nanosilicate reinforced hydrogel with dual-lineage bioactivity for osteochondral tissue engineering. Mater. Sci. Eng. C-Materials Biol. Appl. 127, 112215. 10.1016/j.msec.2021.112215 34225867

[B125] Zhang J. Ebraheim N. Lause G. E. Xiao B. Xu R. (2012a). A comparison of absorbable screws and metallic plates in treating calcaneal fractures: A prospective randomized trial. J. Trauma Acute Care Surg. 72, E106–E110. 10.1097/ta.0b013e3182231811 22439244

[B126] Zhang J. Ebraheim N. Lause G. E. Xiao B. Xu R. (2012b). A comparison of absorbable screws and metallic plates in treating calcaneal fractures: A prospective randomized trial. J. Trauma Acute Care Surg. 72, E106–E110. 10.1097/ta.0b013e3182231811 22439244

[B127] Zhang S. Huang G. Yuan K. Zhu Q. Sheng H. Yu R. (2017). Tanshinone IIA ameliorates chronic arthritis in mice by modulating neutrophil activities. Clin. Exp. Immunol. 190, 29–39. 10.1111/cei.12993 28542869 PMC5588760

[B128] Zhao J. Griffin M. Cai J. Li S. Bulter P. E. M. Kalaskar D. M. (2016). Bioreactors for tissue engineering: An update. Biochem. Eng. J. 109, 268–281. 10.1016/j.bej.2016.01.018

[B129] Zhao Z. Fan C. Chen F. Sun Y. Xia Y. Ji A. (2020). Progress in articular cartilage tissue engineering: A review on therapeutic cells and macromolecular scaffolds. Macromol. Biosci. 20, e1900278. 10.1002/mabi.201900278 31800166

[B130] Zheng X. Smith W. Jackson J. Moran B. Cui H. Chen D. (2016). Multiscale metallic metamaterials. Nat. Mater. 15, 1100–1106. -+. 10.1038/nmat4694 27429209

[B131] Zhou C. Z. Confalonieri F. Jacquet M. Perasso R. Li Z. G. Janin J. (2001). Silk fibroin: Structural implications of a remarkable amino acid sequence. Proteins-Structure Funct. Genet. 44, 119–122. 10.1002/prot.1078 11391774

[B132] Zhou H. Lee J. (2011). Nanoscale hydroxyapatite particles for bone tissue engineering. Acta Biomater. 7, 2769–2781. 10.1016/j.actbio.2011.03.019 21440094

[B133] Zhou S. Huang W. W. Belton D. J. Simmons L. O. Perry C. C. Wang X. Q. (2015). Control of silicification by genetically engineered fusion proteins: Silk-silica binding peptides. Acta Biomater. 15, 173–180. 10.1016/j.actbio.2014.10.040 25462851 PMC4331239

[B134] Zhu W. Ma X. Y. Gou M. L. Mei D. Q. Zhang K. Chen S. C. (2016b). 3D printing of functional biomaterials for tissue engineering. Curr. Opin. Biotechnol. 40, 103–112. 10.1016/j.copbio.2016.03.014 27043763

[B135] Zhu W. Ma X. Gou M. Mei D. Zhang K. Chen S. (2016a). 3D printing of functional biomaterials for tissue engineering. Curr. Opin. Biotechnol. 40, 103–112. 10.1016/j.copbio.2016.03.014 27043763

